# Total flavonoids of rhizoma drynariae improved the osteogenic and pro-angiogenic capacities of BMSCs to accelerate long bone regeneration

**DOI:** 10.1038/s41598-025-19511-8

**Published:** 2025-10-10

**Authors:** Hengjun Huang, Silu Li, Huang Zhan, Fenfang Gong, Jian Liu, Zhenya Liu, Hui Li, Chengyu Yang

**Affiliations:** 1 Jiangxi Province Key Laboratory of Traditional Chinese Medicine Pharmacology, Institute of Traditional Chinese Medicine Health Industry, China Academy of Chinese Medical Sciences, Nanchang, 330115 China; 2 Jiangxi Health Industry Institute of Traditional Chinese Medicine, Nanchang, 330115 China; 3 Institute of Chinese Vateria Medica, China Academy of Chinese Medical Sciences, Beijing, 100700 China

**Keywords:** TFRD, Bone regeneration, BMSCs, Endothelial YAP1/TAZ, HIF-1α signaling pathway, Mesenchymal stem cells, Medical research

## Abstract

Total Flavonoids of Rhizoma Drynariae (TFRD) possess the ability to enhance bone regeneration by promoting angiogenesis-osteogenesis coupling, but the underlying mechanisms remain poorly understood. This study aimed to investigate the influence of TFRD on the coordination of osteogenic and pro-angiogenic properties of bone marrow mesenchymal stem cells (BMSCs) during long bone regeneration and further elucidate the underlying mechanism. We initially assessed pharmacological effects of TFRD in mouse monocortical tibial defect (MTD) model by utilizing micro-CT, histopathology and immunofluorescence imaging. Subsequently, we extracted BMSCs from TFRD-treated mice and characterized the regulatory effects of TFRD on the behavior of BMSCs. We observed that TFRD treatment increased skeletal parameters, osteoblast number, vessel volume and perivascular osteoprogenitor population within the callus. In vitro analyses revealed that TFRD promoted the proliferation and osteogenic differentiation of BMSCs. Additionally, BMSC-conditioned medium from TFRD-treated mice enhanced the migration, proliferation and tube formation of endothelial cells (ECs) under hypoxia. TFRD increased the expression level of HIF-1α and its target genes in callus and hypoxia-cultured ECs. The results of YAP1/TAZ interference in ECs by using siRNA indicated that the enhancement of HIf-1α was through YAP1/TAZ inhibition. Our findings suggest that YAP1/TAZ/ HIf-1α pathways involved in indirect regulation of TFRD on angiogenesis through BMSCs paracrine pathways.

**Supplementary Information:**

The online version contains supplementary material available at 10.1038/s41598-025-19511-8.

## Introduction

Bone defects, arising from external trauma, infections or tumor surgeries, are prevalent conditions encountered in orthopedic clinics. Recent studies indicate that the prevalence of clinical defects has reached 4% among individuals aged 40 years and older in China^1^. Although skeletal tissue can self-regenerate, extensive damage or adverse environmental factors can impede healing, leading to delayed recovery or complete failure regeneration, known as nonunion^1,2^. Clinical evidence suggests that approximately 5–10% of defects do not heal properly and that the mortality rate following defects is increasing^3^. The delayed union and nonunion severely damage patients’ physical functions and place substantial demands on the healthcare system^4^. Therefore, accelerating bone regeneration and preventing nonunion are of great significance in orthopedic practice.

Bone regeneration is a complex biological process involving intricate interactions among multiple cell types, with bone marrow mesenchymal stem cells (BMSCs) playing central regulatory roles^5^. BMSCs contribute to bone matrix formation through direct differentiation into chondrogenic/osteogenic precursor cells, while simultaneously orchestrating the behavior of various repair-associated cells^6,7^. Recent studies suggested that BMSCs could mediate angiogenesis through paracrine mechanisms^8^. Specifically, BMSCs-derived cytokines and exosomes enhance vascular formation by activating endothelial FGFR and VEGFR-dependent signaling pathways in vascular endothelial cells^9–11^. Notably, this neovascularization reciprocally establishes a supportive microenvironment that enhances stem cell viability and osteogenic differentiation potential - a critical regulatory loop termed angiogenesis-osteogenesis coupling^12–14^.

Yes-associated protein 1 (YAP1) and PDZ-binding motif (TAZ) are integral components of the Hippo signaling pathway and play diverse functional roles in regulating angiogenesis across different tissues^15^. Endothelial-specific deletion of YAP1/TAZ has been shown to impair vascularization during embryonic development^16^ retina growth^17^ and tumor pathology^18^ but promotes vessel formation in skeletal tissue^19,20^. Hypoxia-inducible factor 1-alpha (HIF-1α) is a transcription factor that orchestrates the cellular response to fluctuations in oxygen levels and has been identified as being closely linked to both physiological and pathological angiogenesis^21^. Notably, recent studies have shown that YAP1/TAZ negatively regulates bone angiogenesis by suppressing HIF-1α signaling in bone endothelial cells (ECs)^19,20^. These findings imply that pharmacologically inhibiting endothelial YAP1/TAZ signaling may offer therapeutic benefits in enhancing bone angiogenesis.

Total flavonoids from Rhizoma Drynariae (TFRD), bioactive compounds extracted from the Traditional Chinese Medicine herb Rhizoma Drynariae, have been widely utilized for osteoporosis treatment^22^. Mechanistic investigations propose that TFRD exhibits osteoanabolic properties, highlighting its novel therapeutic potential for bone defect management^23,24^. Recent research further demonstrates TFRD-enhanced bone regeneration across diverse conditions, including the induced membrane technique^24^ critical-sized defects^25^ and distraction osteogenesis^26^. While substantial research has characterized TFRD-mediated molecular regulation of mesenchymal stem cell osteogenic differentiation^26–28^ its influence on BMSCs-mediated angiogenesis and the underlying mechanism remains poorly understood.

In this study, we explored the pharmacological effects and mechanism of TFRD in regulating osteogenic and pro-angiogenic behavior of BMSCs during mice long bone regeneration. Our results demonstrate that TFRD not only enhances BMSCs osteogenic differentiation but also potentiates angiogenesis via BMSCs paracrine pathway. Mechanically, TFRD induce BMSCs to secrete angiogenic factors that suppress endothelial YAP1/TAZ expression, thereby triggering HIF-1α pathway activation to drive angiogenesis.

## Results

### TFRD treatment accelerated bone regeneration in MTD mice

LC‒MS was performed to identify the main active compounds of TFRD in both positive and negative modes (Fig. S1). As indicated in Table S1, thirteen major flavonoids found in the TFRD were identified, which aligns with a previous report^29^. Among them, naringin and neoeriocitrin, which have been reported to be bioactive ingredients in TFRD^23^ were detected. The secondary mass spectra and structural formulas of naringin and neoeriocitrin in the TFRD are shown in Fig. S2.

**Fig. 1:**
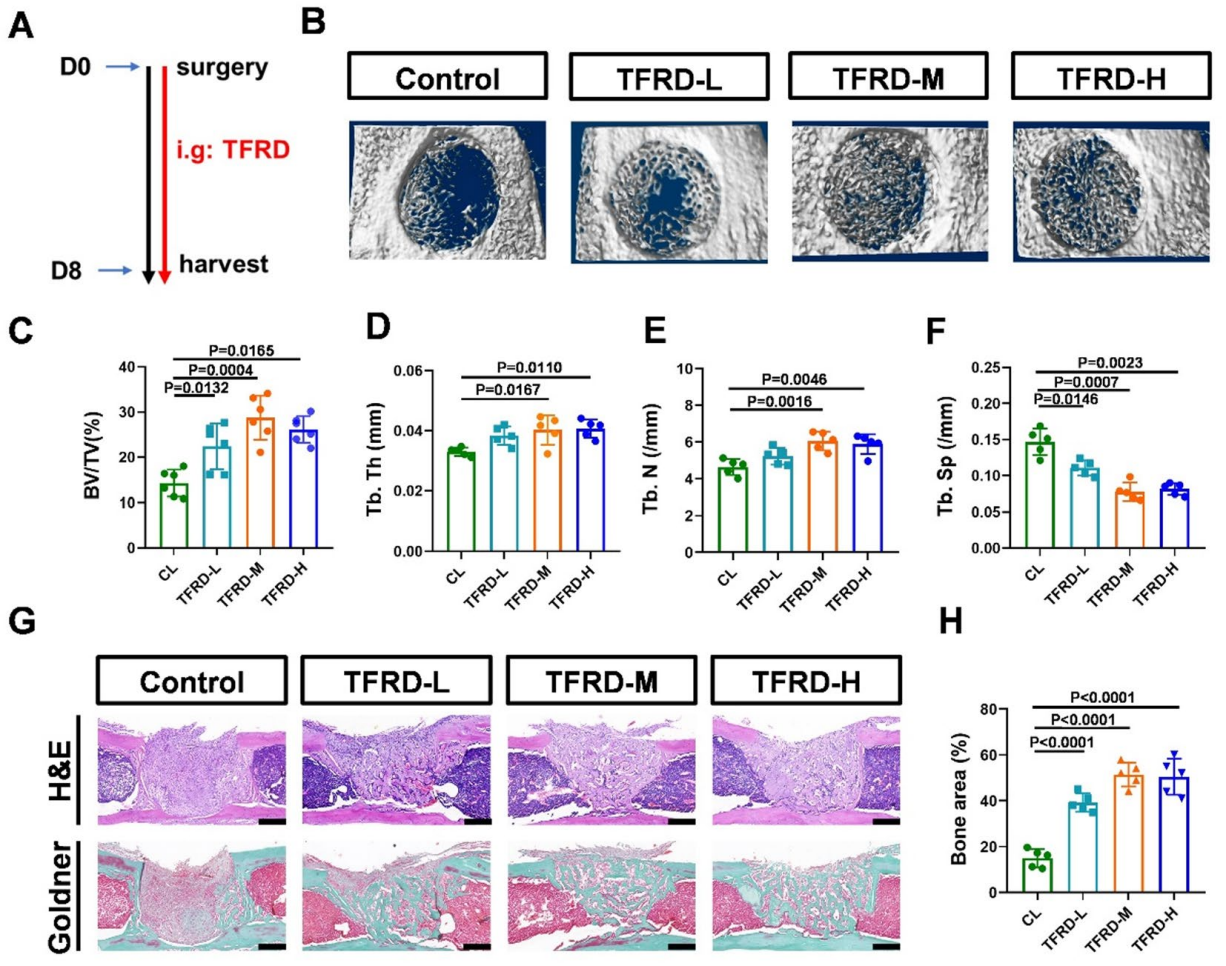
TFRD treatment promoted geometric recovery of newly formed bone in MTD mice. (**A**) Schematic of MTD development and the TFRD administration strategy. (**B**) Representative 3D reconstructions of the bone callus in the control and TFRD treatment groups at PSD 8. Quantitative analysis of the (**C**) BV/TV, (**D**) Tb.Th, (**E**) Tb.N and (**F**) Tb.Sp of the defect region on PSD 8 (*n* = 6). (**G**) H&E and Goldner staining of the bone callus on PSD 8 from each group; scale bar = 100 μm. (**H**) Quantitative analysis of the bone area in the callus on PSD 8; *n* = 6.

To evaluate the pharmacological effects of TFRD on long bone defects, we established an MTD model in mice (Fig. 1A). Micro-CT analysis revealed that, compared with control treatment, TFRD administration induced more bone formation in the callus at PSD8 (Fig. 1B). Compared with the control, middle- and high-dose TFRD treatment significantly increased the BV/TV in the callus. In addition, low-dose TFRD application significantly increased the BV/TV and decreased the Tb. Sp but had no significant effect on the Tb. Th and Tb. N compared with those of the control group (Fig. 1C to F). H&E and Goldner’s trichrome staining results revealed that TFRD application increased bone accrual and mineralized bone in the callus (Fig. 1G and H).

### TFRD treatment promoted osteogenesis during bone regeneration in MTD mice

**Fig. 2:**
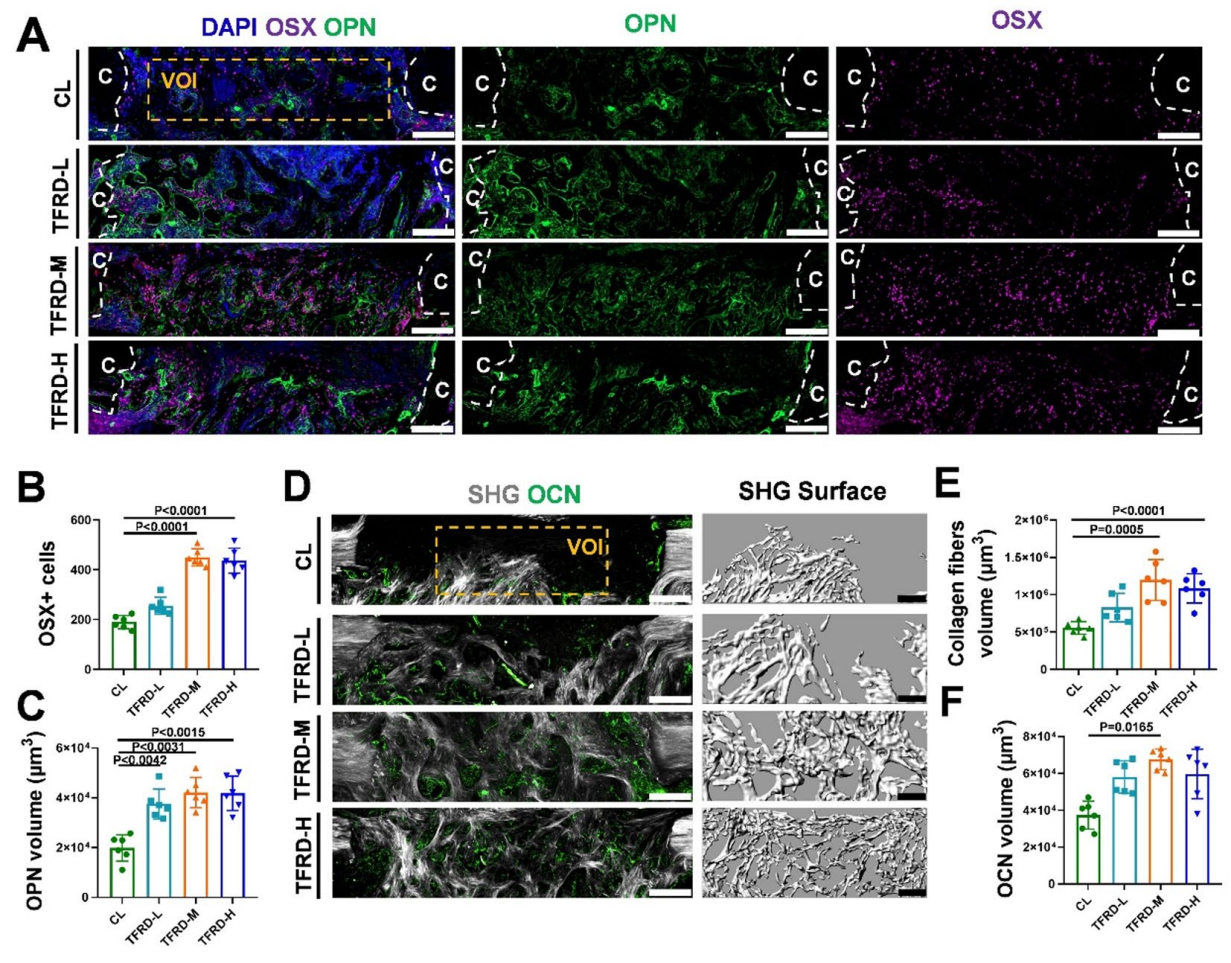
TFRD treatment increased the number of osteoblasts and promoted osteogenic protein expression in callus. (**A**) Immunofluorescence images of OSX (purple), OPN (green) and DAPI (blue) in representative longitudinal thick sections across the MTD defect with and without TFRD treatment on PSD 8; scale bar = 200 μm, c = cortical bone. Quantitative analysis of the number of (**B**) OSX + cells and (**C**) the volume of OPN in the volume of interest (VOI) from A (*n* = 6). (**D**) Confocal and two-photon images of OCN (green) and SHG (gray) in MTD defects with and without TFRD treatment; scale bar = 200 μm. Quantitative analysis of (**E**) SHG volume and (**F**) OCN volume in the VOI from D (*n* = 6).

The population of osteoblasts (OSX+)^30^ and the expression level of osteopontin (OPN), which is essential for osteoblast maturation^31^ were observed via immunofluorescence imaging. The results showed that TFRD application increased the number of OSX + osteoblasts and the expression level of OPN in callus (Fig. 2A to C). We further analyzed the formation of collagen fibers and the expression level of osteocalcin (OCN), a critical component in the process of matrix mineralization^32^. The results revealed that middle and high doses of TFRD increased the volume of collagen fibers in the callus (Fig. 2D and E). The volume of OCNs was also greater in the TFRD-M group than in the control group (Fig. 2F).

### TFRD increased the osteogenic capacity of BMSCs during defect repair

**Fig. 3:**
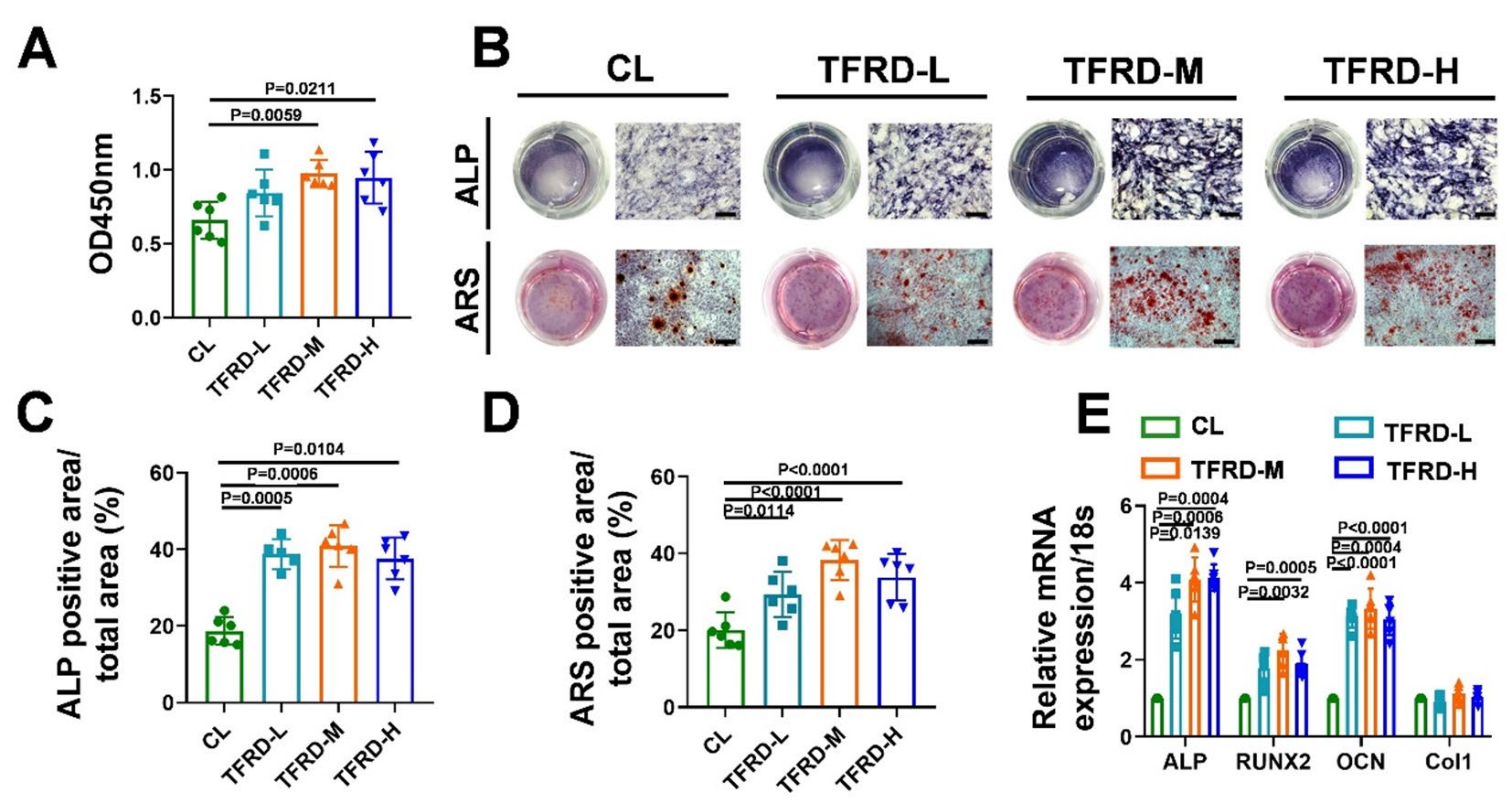
TFRD promoted proliferation and osteogenic differentiation of BMSCs during bone defect repair. (**A**) Effect of TFRD on BMSC proliferation, as demonstrated by a CCK-8 assay (*n* = 6). (**B**) Osteogenic differentiation capacity of control, TFRD-L, TFRD-M and TFRD-H BMSCs on PSD 8, as demonstrated by ALP and ARS staining; scale bar = 100 μm. Quantitative analysis of the proportions of (**C**) ALP-positive and (**D**) ARS-positive areas in A (*n* = 6). (**E**) The expression levels of ALP, RUNX2, OCN and Col1 in the BMSCs from each group at PSD 8 cultured in OG medium for 14 days (*n* = 6).

BMSCs constitute one of the main sources of osteoblastic lineage cells during bone defect repair^33^. To investigate the effect of TFRD on stem cell behavior, we extracted BMSCs from MTD after TFRD treatment. Flow cytometry results showed that the expression of stem cell positive markers (CD29, Sca-1) in the extracted cells was greater than 90%, and the expression of negative markers (CD117, CD31, CD34) was less than 2%, indicating that the extracted cells were BMSCs (Fig. S3). CCK-8 assay revealed that middle- and high-dose TFRD treatment promoted BMSCs proliferation during the bone regeneration (Fig. 3A). Compared with the control treatment, the TFRD treatment resulted in greater ALP activity and more calcium deposition (Fig. 3B to D). QPCR analysis revealed significant increases in the relative expression levels of osteogenic genes (ALP, RUNX2 and OCN) in the TFRD-L, TFRD-M and TFRD-H groups compared with those in the control group (Fig. 3E).

### TFRD enhanced angiogenesis-osteogenesis coupling in the callus

**Fig. 4:**
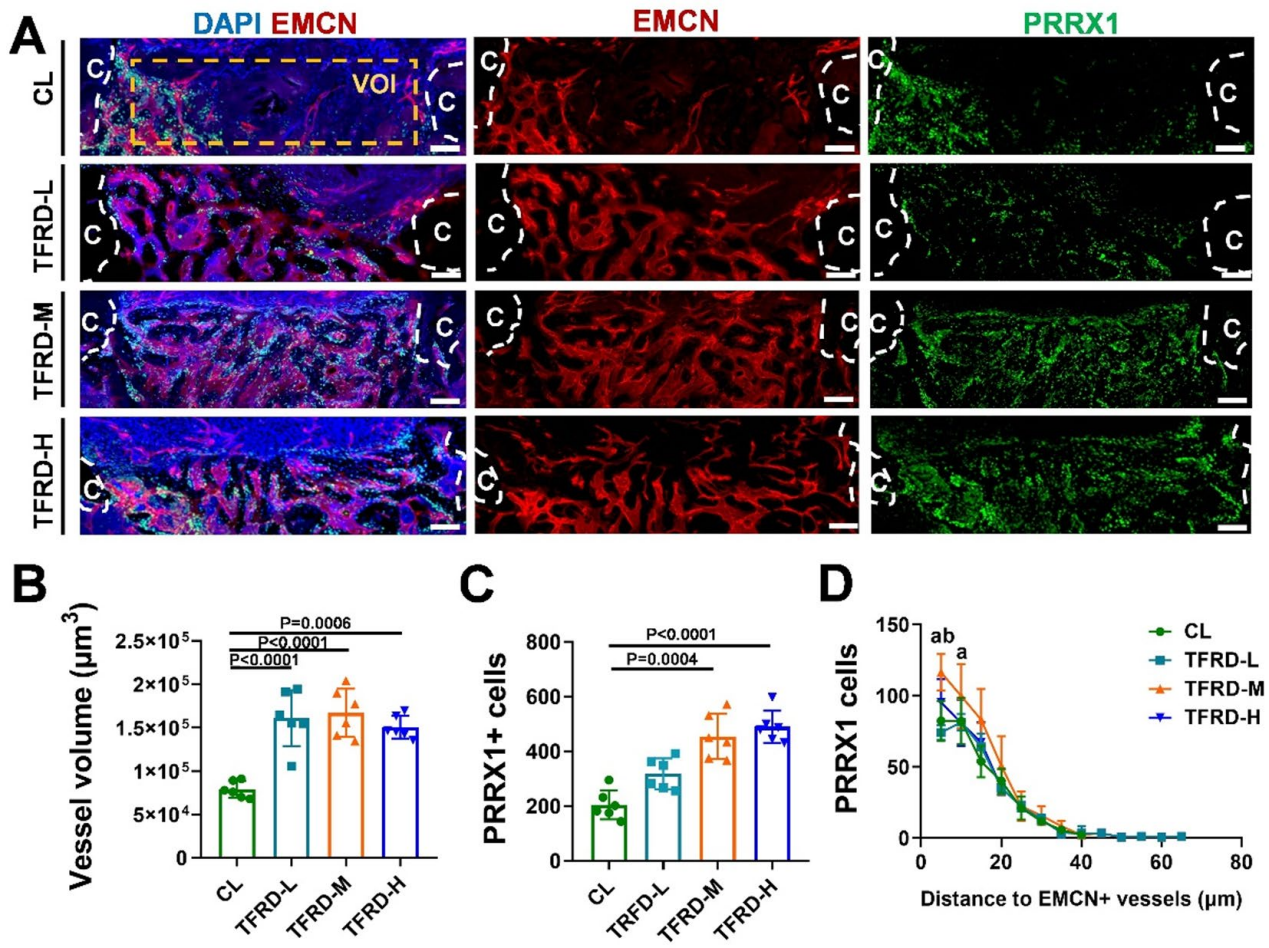
TFRD enhanced angiogenic‒osteogenic coupling during defect repair in MTD mice. (**A**) Immunofluorescence images of EMCN (red), PRRX1 (green) and DAPI (blue) in representative thick longitudinal sections across the MTD defect region at PSD 8; scale bar = 200 μm. Quantitative analysis of (**B**) vessel volume and (**C**) the number of PRRX1 + cells in the VOI from A (*n* = 6). (**D**) Spatial distribution analysis between EMCN + vessels and PRRX1 + osteoprogenitors. Letters “a” indicate a significant difference between TFRD-M and CL, and “b” indicates a significant difference between TFRD-M and TFRD-L (*n* = 6).

We further investigated whether TFRD could enhance angiogenesis-osteogenesis coupling during MTD repair. The newly formed vessels (EMCNs+) and PRRX1 + cells, the periosteal osteoprogenitors which are known to be a major contributor to bone regeneration^34,35^ were measured via deep tissue imaging (Fig. 4A). Compared with those in control group, the vessel volume and PRRX1 + osteoprogenitors population in the TFRD-L, TFRD-M and TFRD-H groups were markedly increased (Fig. 4B and C). Spatial distribution analysis revealed that middle- and high-dose TFRD application significantly increased the number of PRRX1 + osteoprogenitors adjacent to newly formed vessels (Fig. 4D), demonstrating that TFRD may enhance communication between ECs and osteoprogenitors.

### TFRD improved the pro-angiogenic capacities of BMSCs during defect repair

**Fig. 5:**
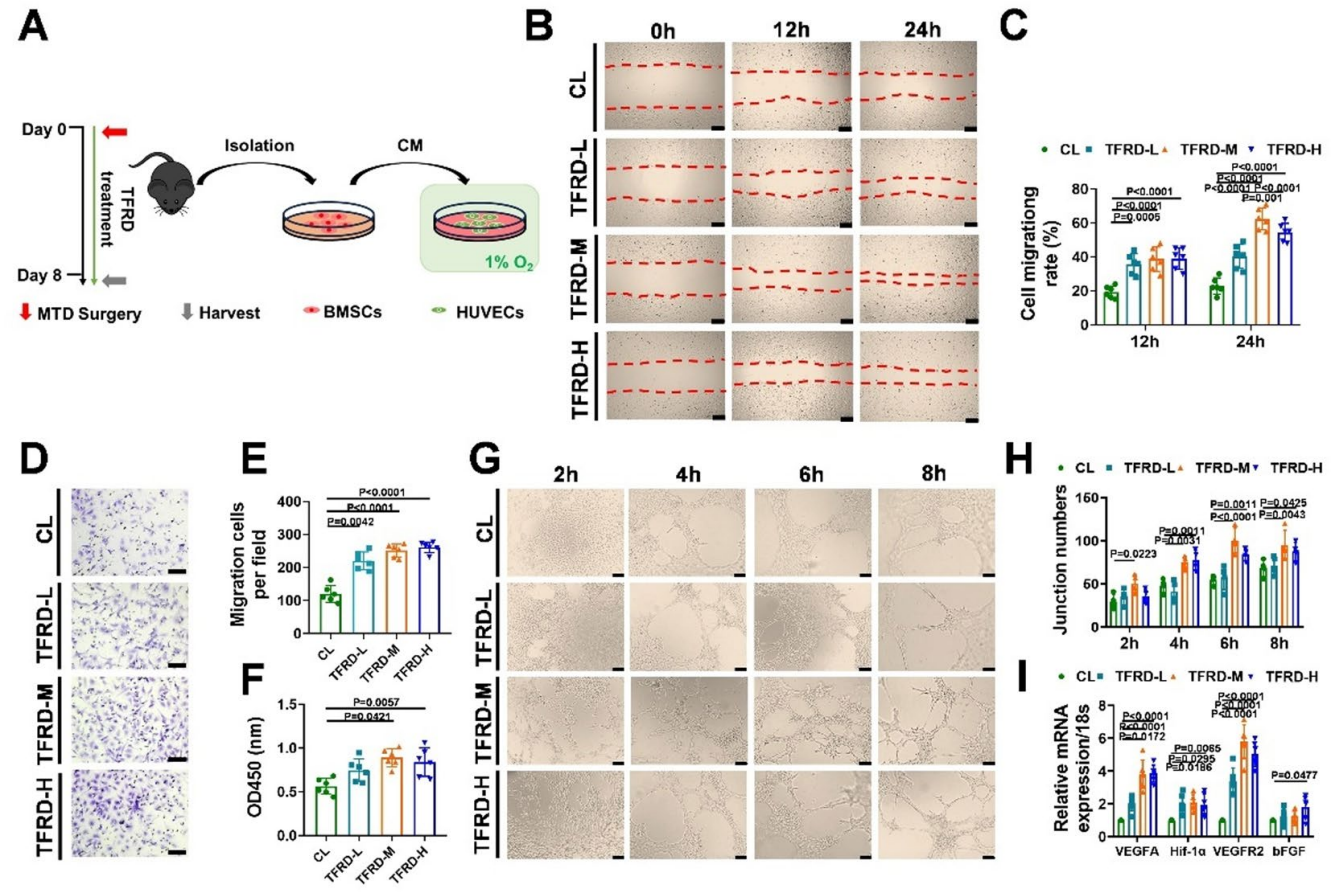
TFRD improved the angiogenic effect of BMSCs-CM. (**A**) Experimental design schematic for the isolation of BMSCs from TFRD-treated MTD donors and the transfer of BMSC-CM into HUVECs. (**B**) Representative images of HUVEC metastasis in the scratch assay; scale bar = 200 μm. (**C**) Quantitative analysis of the percentage of the scratch area in the scratch wound healing assay (*n* = 6). (**D**) Representative images of HUVEC metastasis stained with crystal violet in the transwell assay; scale bar = 100 μm. (**E**) Quantitative analysis of migrated cells in a transwell assay (*n* = 6). (**F**) CCK8 analysis of the effects of BMSC-CM treatment on HUVEC growth (*n* = 6). (**G**) Tube formation of HUVECs in the BMSC-CM-treated groups, which were derived from the CL, TFRD-L, TFRD-M and TFRD-H groups. (**H**) Quantitative analysis of junction numbers in the tube formation assay (*n* = 6). (**I**) The expression levels of angiogenic genes (VEGFA, Hif-1α, VEGFR2 and bFGF) in HUVECs after treatment with BMSC-CM (*n* = 6).

Since the callus possess hypoxic microenvironment^36^we determined the paracrine effects of BMSCs on promoting angiogenesis under hypoxic conditions, and study the regulatory effects of TFRD. The BMSCs were extracted from TFRD treated mice and conditioned medium from BMSCs (BMSCs-CM) were collected to induce cultured HUVECs (Fig. 5A). In the scratch assay, HUVECs in the TFRD-L, TFRD-M and TFRD-H groups exhibited greater migration ability than in the control group (Fig. 5B and C). The transwell assay also revealed that there were more migrated cells in the TFRD groups than in the control group (Fig. 5D and E). CCK8 assay showed that BMSCs-CM from TFRD-treated mice promoted the proliferation of HUVECs (Fig. 5F). The tube formation assay revealed that, compared with the control group, the TFRD groups had more endothelial tubules and more junction formation (Fig. 5G and H). Moreover, the relative mRNA expression levels of proangiogenic genes, including VEGFA, Hif-1α, VEGFR2 and bFGF, in the TFRD-L, M and H groups were greater than those in the control groups (Fig. 5I).

### TFRD inhibited YAP1/TAZ expression and promoted HIF-1α expression in endothelial cells under hypoxic conditions through regulating the paracrine pathway of BMSCs

**Fig. 6:**
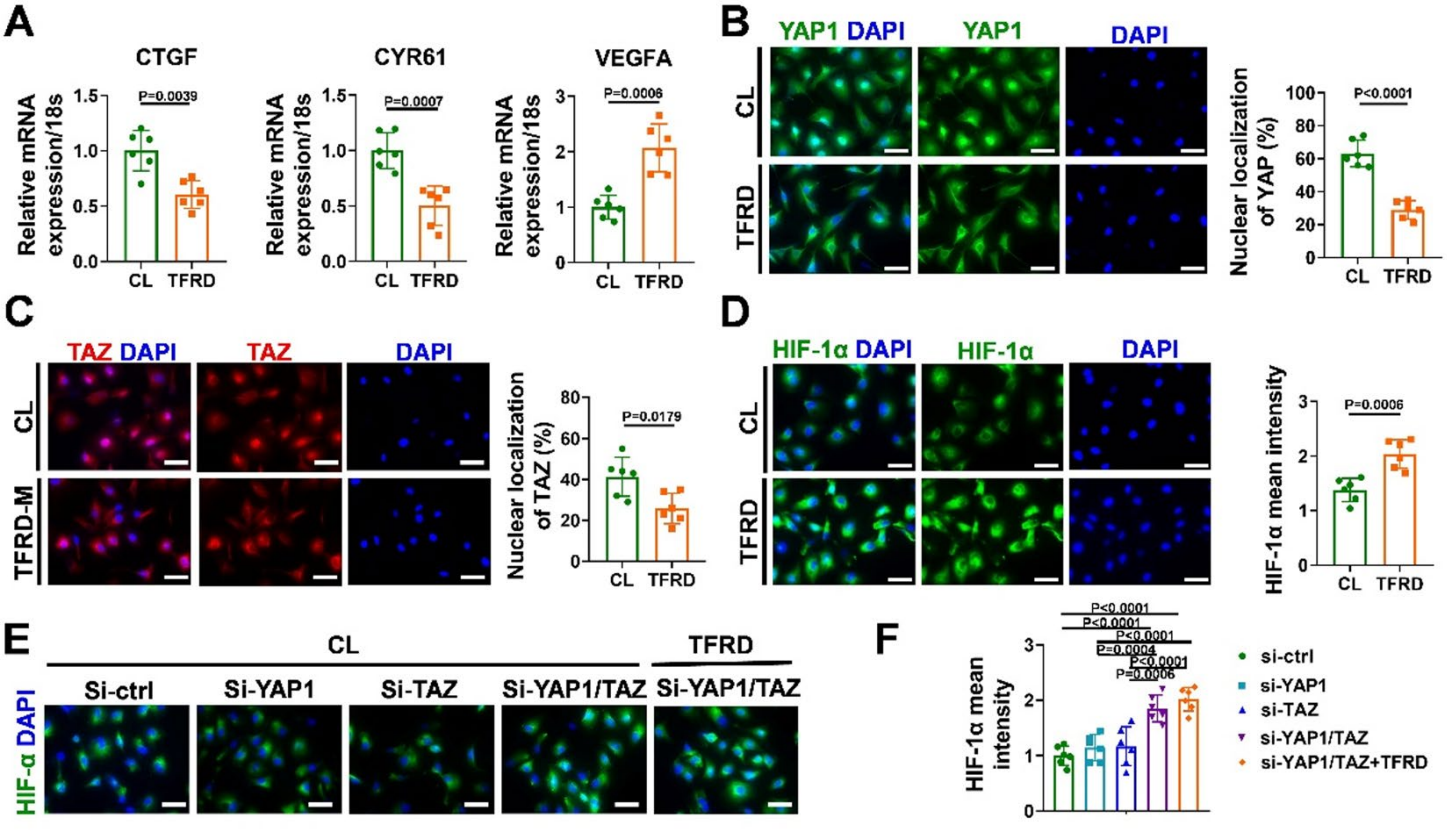
BMSCs-CM from TFRD-treated mice improved HIF-1α expression by inhibiting YAP1/TAZ in ECs under hypoxic conditions. (**A**) The expression levels of the YAP1/TZA target genes CTGF and CYR61 and the HIF-1α target gene VEGFA in HUVECs induced with BMSC-CM from TFRD-treated mice compared with those in control mice under hypoxic conditions (1% O_2_) (*n* = 6). Immunofluorescence images and quantitative analysis of (**B**) YAP1, (**C**) TAZ and (**D**) HIF-1α in HUVECs induced with BMSC-CM from TFRD-treated or saline-treated mice under hypoxic conditions; scale bar = 100 μm. (**E**) Immunofluorescence images of HIF-1α in HUVECs from the si-ctrl, si-YAP1, si-TAZ, si-YAP/TAZ and si-YAP/TAZ + TFRD groups (*n* = 6); scale bar = 100 μm. (**F**) Quantitative analysis of the mean intensity of HIF-1α in HUVECs from each group under hypoxic conditions (*n* = 6).

HIF-1α is a key regulator of the hypoxia response in vascular ECs and has been shown to enhance angiogenesis-osteogenesis coupling in bone^37^. Previous studies have shown that the activation of YAP1/TAZ leads to a reduction in HIF-1α expression under hypoxic conditions, thus negatively controlling bone angiogenesis^19^. We then determined whether HIF-1α signaling and YAP1/TAZ activation involved in TFRD induced angiogenesis. Compared with the control, BMSCs-CM from TFRD-treated mice significantly decreased the expression levels of the YAP1/TAZ target genes CTGF and CYR61, but increased the expression level of the HIF-1α target gene VEGFA (Fig. 6A). The immunofluorescence results revealed BMSCs-CM from TFRD-treated mice decreased the proportion of nuclear YAP1/TAZ (Fig. 6B and C). Additionally, the expression level of HIF-1α was increased in the TFRD group (Fig. 6D).

We further interfered with the expression of YAP1 and TAZ in HUVECs via siRNA. qPCR analysis revealed that siYAP1#1 and siTAZ#2 had the greatest inhibitory effects on target genes in HUVECs and were chosen for subsequent experiments (Fig. S4A and B). Compared with that in the control group, the individual knockdown of YAP1 and TAZ had no effect on the expression level of HIF-1α under hypoxic conditions, but it was significantly greater in the YAP1 and TAZ double-knockdown group, and BMSC-CM from TFRD-treated mice did not further increase this effect (Fig. 6E and F).

### TFRD activated HIF-1α signaling and inhibited YAP1 expression during defect repair in MTD mice

**Fig. 7:**
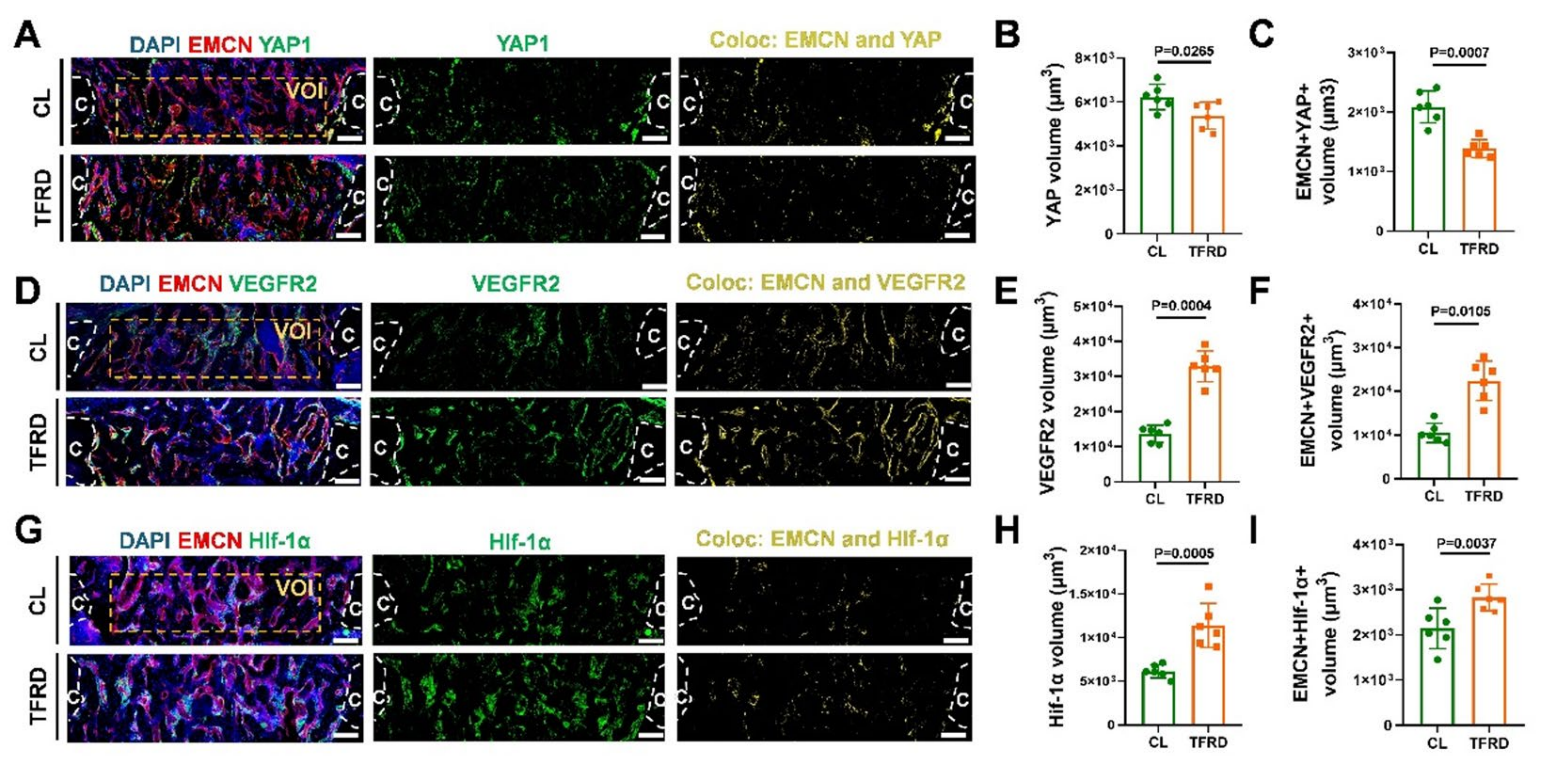
TFRD enhanced HIF-1α signaling by inhibiting endothelial YAP1 expression during MTD repair. (**A**) Representative fluorescence images of EMCN (red), YAP1 (green) and DAPI (blue) in the callus on PSD 8; color: EMCN + YAP + cells; c: cortical bone. Quantitative analysis of (**B**) YAP1 volume and (**C**) EMCN + YAP1 + volume in the callus (*n* = 6). (**D**) Representative fluorescence images of EMCN (red), HIF-1α (green) and DAPI (blue) in the callus at PSD 8; color: EMCN + HIF-1α + cells; c: cortical bone. Quantitative analysis of (**E**) HIF-1α volume and (**F**) EMCN + HIF-1α + volume in the callus (*n* = 6). (**G**) Representative fluorescence images of EMCN (red), VEGFR2 (green) and DAPI (blue) in the callus on PSD 8; color: EMCN + VEGFR2 + cells; c: cortical bone. Quantitative analysis of (**H**) VEGFR2 volume and (**I**) EMCN + VEGFR2 + volume in the callus (*n* = 6).

We further investigate whether TFRD could modulate YAP1 expression and HIF-1α signaling in ECs during bone defect repair. Immunofluorescence images showed that TFRD administration decreased YAP1 expression (Fig. 7A to C), but increased the volume of HIF-1α in callus and newly-formed vessels (EMCN+) (Fig. 7D to F). The d expression of VEGFR2, a downstream component of HIF-1α signaling, was also increased in both the callus and newly formed vessels after TFRD application (Fig. 7G to I).

## Discussion

**Fig. 8:**
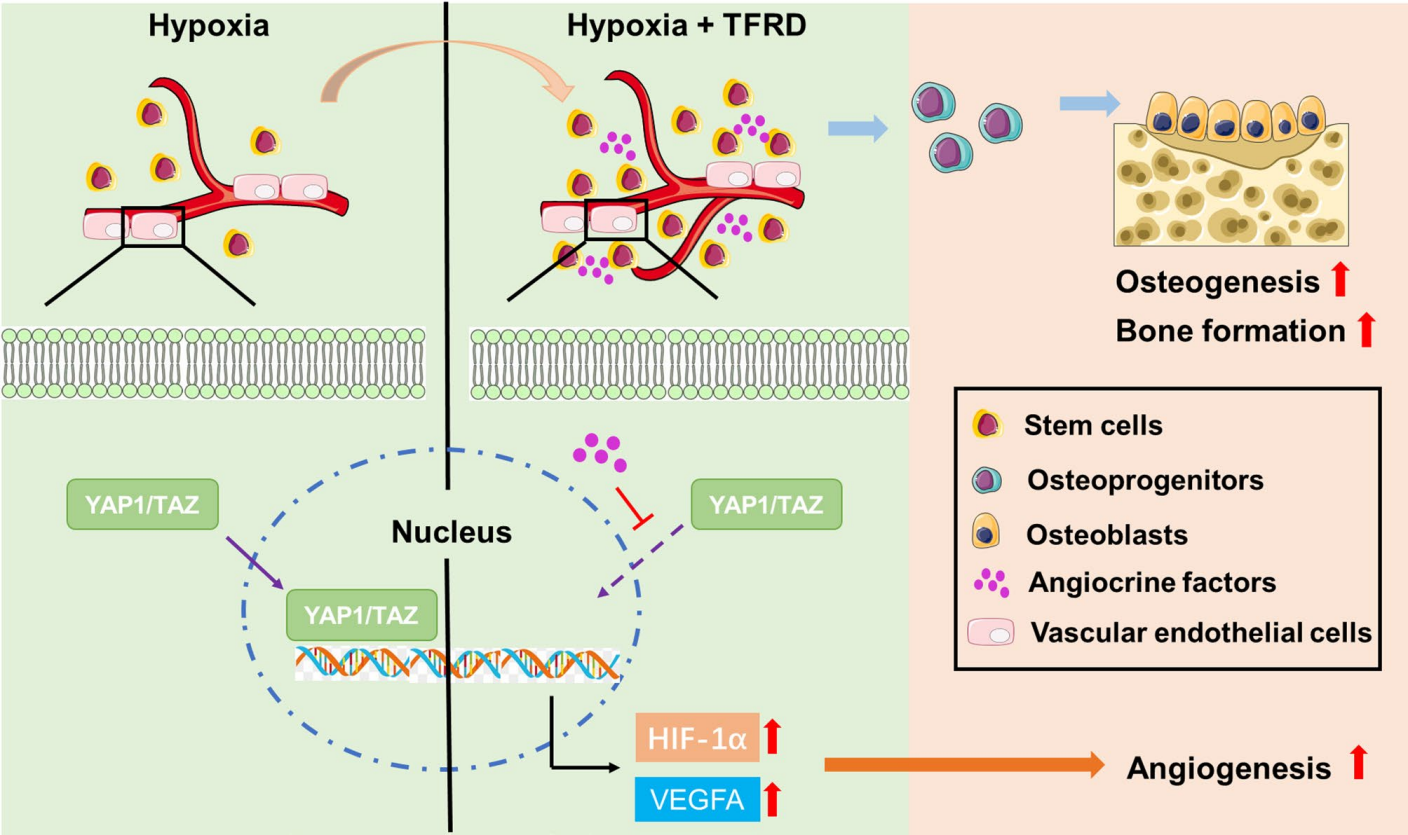
Schematic diagram of the mechanism by which TFRD promote osteogenic and pro-angiogenic capacities of BMSCs during bone defect repair.

Bone fracture healing is a complex and dynamic process that relies on numerous cell types and the interactions of various systems. Although skeletal tissue has self-repair capacity, approximately 10% of long bone fractures cannot be successfully repaired because of excessive area, interference from the external environment, etc., resulting in bone nonunion^2^. TFRD, a total flavonoid extracted from the traditional Chinese medicine Rhizoma Drynariae, was developed as a post-marketing Chinese medicine and is widely used to treat osteoporosis^38^. Recent studies have demonstrated that TFRD also has beneficial effects on bone defect repair^25,26^ but the underlying mechanism needs to be further excluded. In this study, we found that TFRD enhanced osteogenic and pro-angiogenic capacities of BMSCs, thus accelerating bone regeneration in MTD mice. Further mechanistic experiments revealed that TFRD could promote angiogenesis through regulating stem cell paracrine pathways. After TFRD treatment, stem cell-derived paracrine factors promote HIF-1α expression through the inhibition of YAP1/TAZ signaling in ECs, thus promoting angiogenesis during bone defect repair (Fig. 8). These findings may broaden the potential clinical applications of TFRD and provide a new therapeutic strategy for the treatment of bone defects.

TFRD has been developed into the post-marketing Chinese medicine (Qianggu capsule) for utilizing in clinical practice to treat osteoporosis^38,39^. Recently, oral administration of TFRD to promote bone defect healing has been demonstrated in animal models^25,40^. However, most of these trials have focused on the effect of TFRD on endochondral ossification, and few reports have focused on the pharmacodynamic effect of TFRD on intramembranous ossification of long bones. Intramembranous ossification, such as distraction osteogenesis-induced long bone defect treatment, involves the direct differentiation of mesenchymal stem cells to osteoblasts and the construction of a bone matrix^41^. Here, we established a mice MTD model, which produces a consistent pattern of angiogenesis and revascularization in addition to bone matrix deposition^42–44^ and systematically investigated the role of TFRD in intramembranous ossification during long bone regeneration. We found that TFRD accelerated bone repair in MTD mice, as evidenced by increased BV/TV, Tb.Th, Tb.N and bone matrix deposition.

Osteoblasts are crucial for bone defect repair, as they are major cells that can give rise to bones^30^. Thus, an increase in the osteoblast population is beneficial for accelerating bone defect repair. However, because of their limited life span, osteoblasts must be replenished by upstream cells, including skeletal stem cells, osteoprogenitors and preosteoblasts^45,46^. In this study, we found that TFRD increase the population of oblasts (OSX+) and promote the secretion of osteogenic-related proteins (OCN and OPN) in callus. The beneficial effect of TFRD on promoting osteogenic differentiation of BMSCs has been established^47^. However, the majority of these studies performed TFRD to induce BMSCs in vitro, and limited research addressing the impact of oral administration of TFRD on BMSCs in vivo. By extracting BMSCs on PSD 8, we found TFRD treatment increased potential of osteogenic differentiation and proliferation of BMSCs, demonstrating the multi-dimensional enhancement of TFRD on BMSCs osteogenic capacities. While this study prioritized core osteogenic markers across differentiation phases, future investigations would benefit from extended profiling including BSP and OPN to further resolve terminal maturation stages. In addition, both periosteal- and bone marrow-derived stem cells/osteoprogenitors contribute to MTD regeneration process^43^. We found that TFRD also enhanced the population of PRRX1 + cells, which were identified as the primary periosteal osteoprogenitors^34,35^ demonstrating that TFRD also show beneficial effect on the behavior of periosteal stem cell.

Skeletal tissue is a highly vascularized system. During bone regeneration, newly formed vessels not only deliver oxygen, hormones and necessary nutrients but also secrete specific cytokines to regulate the behavior of perivascular cells^48^. The ability of TFRD to promote angiogenesis has been identified in skeletal tissue, but exploration of the underlying mechanism has focused mainly on the direct effect of TFRD on the behavior of vascular ECs^24,27^. Recent studies have shown that skeletal stem cells can mediate angiogenesis through paracrine pathways^49^. In this study, we found that TFRD application improved the pro-angiogenic capacity of BMSCs in a hypoxic state, which is representative of the environment in the callus^50^. In vitro results showed that BMSCs-CM from TFRD-treated mice improved various steps of angiogenesis under hypoxia, including the proliferation, migration, and tube formation. These data suggest that the beneficial effect of TFRD on angiogenesis involves not only direct pathways but also indirect pathways through the regulation of the BMSCs paracrine pathway.

The transcriptional coactivator YAP1/TAZ has been shown to play a critical role in sprouting angiogenesis. Deletion of YAP1/TAZ in ECs leads to vascular malformation and barrier disruption in retinal angiogenesis^51–53^. However, recent studies revealed that YAP1/TAZ activation negatively affects skeletal angiogenesis in both normal and pathological states^19,20^. During skeletal development and fracture healing, endothelial YAP1/TAZ suppressed vascular formation and bone formation by inhibiting the classic HIF-1α/VEGF/VEGFR2 signaling pathway^19,20^. A recent study reported that the administration of TFRD increased the angiogenic activity of cultured ECs through the PDGF-BB/PBGFR-β pathway^26^. In this study, we found that BMSC-CM from TFRD-treated mice increased the expression of HIF-1α and its downstream proteins, VEGFA and VEGFR2. Our in vitro experiments showed individual knockdown of YAP1 and TAZ had no effect on the expression level of endothelial HIF-1α under hypoxic conditions, which may due to feedback regulation and compensation mechanism of YAP1/TAZ^54,55^. Kishor et al. reported that YAP1/TAZ could bind with HIF-1α under hypoxic conditions, resulting in the inhibition of its activity^54^. Our findings indicate that BMSC-CM derived from TFRD-treated mice did not further enhance the expression of endothelial HIF-1α in subjected to YAP1/TAZ double knockdown group. This observation suggests that the upregulation of HIF-1α may be attributable to the suppression of YAP1/TAZ expression. Our in vitro and in vivo results confirmed that the HIF-1α signaling pathway is activated partly by the inhibition of YAP1/TAZ in ECs during bone regeneration. These results reveal that TFRD regulates endothelial YAP1/TAZ and expand the understanding of the mechanism by which TFRD promotes the healing of bone defects.

In summary, this study highlights the significant therapeutic potential of TFRD in bone defect healing. Our findings demonstrate that TFRD not only effectively promote osteogenesis, but also improve angiogenesis by regulating the paracrine pathway of BMSCs. In mechanism, TFRD treatment promotes the angiocrine release of BMSCs, which inhibits endothelial YAP1/TAZ expression and further activates the HIF-1α/VEGF/VEGFR2 signaling pathway. These results indicate that TFRD could represent a promising therapeutic agent for bone defect healing.

One limitation in our study is the lacking of clarity regarding the specific bioactive substances contributes to indirect effect of TFRD-induced angiogenesis through regulating stem cell paracrine pathways. Stem cells could indirectly activate endothelial cells by secreting exosomes and bioactive molecules, such as VEGFA, PDGF-BB, Ang-2, etc^56^. Therefore, we hope future perform RNA-seq, siRNA and antibody blocking to identify the key bioactive factor involved in TFRD-mediated indirect angiogenesis. The other limitations of this study is that we did not use multiple methods to detect the protein of interest, and the single immunofluorescence quantification method affected the accuracy of our conclusions.

## Materials and methods

### Reagents

The TFRD used in this study was purchased from Beijing Qihuang Pharmaceutical Manufacturing Co., Ltd. (QiangGu capsule, 0.25 g per capsule; National Approval No. Z20030007; Item No. 04080081; each capsule contained 0.18 g of TFRD). CCK8 and ALP kits were purchased from Beyotime Biotechnology Co., Ltd. (Shanghai, China). Alizarin Red S solution was acquired from Solarbio Life Science Co., Ltd. (Beijing, China). TRIzol reagents, RevertAid First Strand cDNA Synthesis Kit, and GoTaq^®^ qPCR Master Mix were acquired from Thermo Fisher Scientific Co., Ltd. (Waltham, USA).

### Animal model of monocortical tibial defect (MTD)

Male C57BL/6J mice (20 ± 2 g in weight, 8–10 weeks old) were purchased from SPF Biotechnology Co., Ltd. (Beijing, China). License number: SCXK 2019-0010). The mice were kept in the animal facility with a 12 h light/dark cycle and were provided food and water. The MTD model was established as previously reported^42^. Briefly, the mice were anesthetized via inhalation of isoflurane. After the skin was incised and the bilateral tibia of each mouse was exposed, 1 mm diameter circular defects were created on the anterior medial surface of the bilateral tibias of the mice via a high-speed drill at 23000 rpm. The defect center was located 4.3 mm below the proximal articulating surface of the proximal tibia. The muscle and skin were sutured with 5–0 nylon sutures and covered with erythromycin ointment. All animal experiments were approved by the Committee on Animal Care and Use of the Institute of Traditional Chinese Medicine Health Industry, China Academy of Chinese Medical Sciences (Animal Ethics Committee approval number: 2024008). All experiments were performed in accordance with relevant guidelines and regulations. All studies were carried out in accordance with ARRIVE guidelines.

### TFRD administration

After being weighed, the mice were randomly divided into 4 groups (*n* = 6 for each group): the control group (CL) and the low-, middle- and high-dose TFRD groups (TFRD-L, TFRD-M, and TFRD-H, respectively). The medication treatment started on the day of surgery. The mice in the TFRD-L, TFRD-M and TFRD-H groups were administered TFRD at 0.11, 0.22 and 0.44 g/kg/day (i.g.), respectively. The equivalent dose of TFRD was calculated according to the body surface area and based on previous reports^57,58^. The mice in the CL group were given the same volume of normal saline (i.g.). In all the experiments, the mice were euthanized using CO_2_, followed by cervical dislocation on post-surgery day (PSD) 8. All the tibias and BMSCs were collected.

### Micro-computed tomography (micro-CT) analysis

The fresh tibia was dissected and scanned in a SkyScan 1172 high-resolution scanner (Bruker, USA) at a 6 μm resolution with a voltage of 60 kV and a current of 100 µA. The images were captured at 2000 × 1332 pixels with 0.25-degree rotation steps over 180 degrees, two frame averages, and a 0.5-mm Al filter. The densities of the phantoms are 0.25 g/cm^3^ and 0.75 g/cm^3^, respectively.

The defect region was reconstructed via Mimics (Materialise, USA) software. The volume of interest (VOI) in the callus was segmented from a circular region parallel to the surface of the anteromedial region of the tibia and extended 200 μm toward the bone marrow to create a cylindrical volume. The 3D reconstruction of the VOI was performed with Mimics 17 (Materialise, USA), and the total bone volume (BV), total tissue volume (TV), percent bone volume (BV/TV), trabecular number (Tb.N), trabecular thickness (Tb.Th) and trabecular separation (Tb.Sp) were quantified via CTAn software (Bruker, Germany).

### Histological assessment

Fresh tibias were fixed in 4% paraformaldehyde at 4 °C for 24 h and decalcified in 0.5 M EDTA at 4 °C for 21 days. The EDTA solutions were changed every 3 days. The tibias were then dehydrated in gradient alcohol, embedded in paraffin and cut into 5 μm-thick sections. After baking at 68 °C, three sections of each sample were stained with hematoxylin and eosin (H&E) (G1120, Solarbio, China) or Goldner staining solution (G3550, Solarbio, China) according to the manufacturers’ instructions.

### Immunofluorescence staining

Fresh tibiae were fixed in 4% paraformaldehyde at 4 °C for 4 h and decalcified in 0.5 M EDTA at 4 °C for 24 h. The tibiae were cryoprotected in 20% sucrose solution at 4 °C for 24 h, embedded in gelatin-based medium and stored at -80 °C. Tibiae were cryosectioned into 80 μm thick tissue slices along the coronal plane via a cryostat (Leica CM1950, Weztlar, Germany). The samples were stained overnight with the following primary antibodies: anti-Osterix (1:200; ab22552, Abcam, Cambridge, UK), anti-Prrx1 (1:200; ab211292, Abcam, Cambridge, UK), anti-OPN (1:200; ab75285, Abcam, Cambridge, UK), anti-OCN (1:200; ab133612, Abcam, Cambridge, UK), anti-Endomucin (1:200; sc-65495, Santa Cruz Biotechnology, USA), anti-YAP1 (1:200; PA5-87568, Thermo Fisher Scientific, USA), anti-HIF-1α (1:200; ab179483, Abcam, Cambridge, UK), and anti-VEGFR2 (1:200; MA5-15157, Thermo Fisher Scientific, USA). After washing three times with PBS, Alexa Fluor secondary antibodies from donkey (1:400; Thermo Fisher Scientific, Waltham, MA, USA) were added to the samples for staining for 90 min. The slides were mounted with DAPI-Fluorom-G (0100–20, SouthernBiotech, Birmingham, USA) and coverslipped. The staining specificity was confirmed through parallel negative controls, in which the primary antibodies were removed and only added the secondary antibody to the sample (Fig. S5).

### Confocal and two-photon imaging

Three-dimensional fluorescence images were acquired with a 20× objective lens with a Nikon A1R confocal microscope (Tokyo, Japan). Z-stacks of 100 μm in thickness were taken at a size of 1024 × 1024 pixels and an x-y resolution of 0.624 mm with a z-step of 2 μm. The 1-mm defect is imaged by tiling three Z-stacks, spanning 1500 mm along the long axis of the tibia, from one side of the intact cortical bone to the other. DAPI images were acquired (425–475 nm filter) with 405 nm excitation, EMCN images were acquired (550–615 nm filter) with 594 nm excitation. PRRX1, OPN and OCN were acquired (510–540 nm filter) with 488 nm excitation. OSX were acquired (660–735 nm filter) with 647 nm excitation. The scanning speed is 200 frames/second.

The second harmonic generation (SHG) of collagen fibers was acquired on an Olympus FVMPE-RS Multiphoton Laser Scanning Microscope (Japan). The images were excited with an 860 nm laser, and the emission was detected via 420–465 filters. A z-stack 40 μm in height and an x-y detection area 1024 × 1024 pixels in size with a resolution of 0.623 μm were taken for each sample. This volume of interest is the central region of the tibial defect. A successful repair process results in bone formation in this volume.

### BMSCs isolation and BMSCs-CM collection

BMSCs were extracted from mouse femurs according to previously reported methods^59^. In brief, the mice were sacrificed and soaked in 75% ethanol. The skin and muscle surrounding the bone were removed and kept on ice in PBS containing 1% penicillin/streptomycin until further dissection. A 1-ml syringe filled with growth medium (DMEM/F12 + 10% FBS + 1% penicillin/streptomycin) was inserted into the marrow cavity. The effluent was passed through a 200-mesh filter and incubated with RBC lysis buffer (420301, Biolegend, California, USA) to remove red blood cells. After certification, the cells were cultured in growth medium for 3 passages to harvest the BMSCs. According to previous reported^59^BMSCs were identified by labeling with CD29, CD44, Sca-1, CD117 and CD31 fluorescent conjugated antibodies. The results of evaluation are displayed in Supplementary Fig. 4.

For the preparation of bone marrow stem cell-conditioned medium (BMSC-CM), the culture medium was replaced with serum-free essential medium. The cells were then incubated for 72 h. Following this incubation period, the medium was collected and centrifuged at 1500 rpm for 5 min at 4 °C. The resulting supernatants were subsequently centrifuged again at 3000 rpm for 3 min at 4 °C. The final supernatants were filtered via a 0.22 μm filter unit and stored in small tubes at -80 °C until use in the study.

### Alkaline phosphatase (ALP) and Alizarin red S (ARS) staining

The osteogenic capacities of the BMSCs were measured via ALP and ARS staining. In brief, BMSCs from each group were seeded in 24-well plates (5 × 10^4^ cells per well) and cultured in complete culture medium (DMEM/F12 with 10% FBS and 1% penicillin/streptomycin) at 37 °C in a humidified 5% CO_2_ incubator. After overnight attachment, the medium was changed to osteogenic medium (F12 with 10% FBS, 1% penicillin/streptomycin, 50 µg/ml ascorbic acid, 10 mM sodium β-glycerophosphate and 1 µM dexamethasone). After 7 days of osteogenic induction, ALP staining was performed by using a BCIP/NBT alkaline phosphatase color development kit (C3206, Beyotime, Shanghai, China) following the manufacturers’ instructions. After 21 days of induction, ARS staining was performed by using Alizarin Red S staining solution (G1450, Solarbio, Beijing, China).

### Cell migration assay

Cell migration was evaluated by performing transwell and wound healing assays. Human umbilical vein endothelial cells (HUVECs, Sunncell, China) were cultured in endothelial growth medium (DMEM + 10%FBS + 1% penicillin/streptomycin) in 37 °C incubator with 5% CO_2_. HUVECs were passaged when the monolayer confluence reached 70–80%. Subsequent experiments employed cells from passages 3–6.

For the transwell assay, HUVECs were seeded onto the upper layer of the transwell with an 8 μm membrane (3412, Corning, USA) at a density of 2 × 10^4^ cells per well. The lower chamber contained 400 µl of induction medium, which contained BMSC-CM and complete medium at a 1:1 ratio. Induction medium was applied only once during the experiment. After 12 h of incubation under hypoxic conditions (1% O_2_ and 5% CO_2_ at 37 °C), the cells on the front of the upper chamber were gently removed, while the migrated cells on the reverse were stained with 0.1% crystal violet (G1063, Solarbio, Beijing, China) for 30 min before observation. For the wound healing assay, HUVECs were seeded into 12-well plates and cultured in endothelial growth medium. When grown to a monolayer confluence of 70–80%, the cells were scratched with a 1 ml sterilized micropipette tip, the detached cells were removed by washing with PBS. Then applied incubation medium and the samples were incubated under hypoxic conditions (1% O_2_ and 5% CO_2_ at 37 °C). The wound sizes at 0 h, 12 h and 24 h were recorded with a microscope, and the migration rate was calculated via ImageJ software.

### Tube formation assay

Before the tube formation assay, the medium of the HUVECs was replaced with starvation medium (DMEM with 1% FBS and 1% PS), and the HUVECs were cultured for 24 h. Twenty-four-well plates were coated with 100 µl of Matrigel (356234, BD Biosciences, USA) and incubated for 30 min to polymerize before use. Mix approximately 300 µl of BMSC-CM with resuspended HUVECs (1 × 10^5^ cells/ml) and seeded into each well and cultured under hypoxic conditions (1% O_2_ and 5% CO_2_ at 37 °C). After 2 h, 4 h, 6 h and 8 h of incubation, the tube-like structures were imaged via an inverted microscope and the total branching length were analyzed by Image J software.

### RNA interference

siRNAs targeting YAP1 (si-YAP1 #1, 2, and 3) and TAZ (si-TAZ #1, 2, and 3) were purchased from GenePharma (Shanghai, China), and the target sequences are listed in supplementary Table 2. The transfection process was performed by using the GP-transfect-Mate (G04009, GenePharma, Shanghai, China) regent according to the manufacturer’s instructions. Briefly, when HUVECs reached 60–70% confluence, the GP-transfect-Mate reagent was mixed with si-YAP1/TAZ in serum-free DMEM and added to the cells. After incubation for 48 h, the inhibitory efficiency was measured via qPCR analysis. The most effective siRNAs were used for further experiments.

### Immunocytochemistry

HUVECs were cultured in Glass coverslip. After the experiment, cells were rinsed with PBS and fix with 4% paraformaldehyde for 15 min at room temperature. Permeabilize cells with 0.2% Triton X-100 in PBS for 10 min, followed by blocking in 5% for 1 h at room temperature. The cells were incubated overnight with the following primary antibodies: anti-YAP1 (1:500; PA5-87568, Thermo Fisher Scientific, USA), anti-TAZ (1:500, HPA007415, Sigma-Aldrich, USA) and anti-HIF-1α (1:500; ab179483, Abcam, UK). Following PBS washes, cells were incubated with Alexa Fluor 488 secondary antibodies from donkey (1:500; Thermo Fisher Scientific, USA) for 1 h. Coverslips were taken out and mounted with DAPI-Fluorom-G (0100–20, SouthernBiotech, Birmingham, USA). Images were acquired using a confocal microscope with 40× oil objective. Z-stacks (0.5-µm intervals) were processed for maximum-intensity projections.

### QPCR

Total RNA from HUVECs and BMSCs was extracted with TRIzol reagent (15596, Thermo Fisher Scientific, Waltham, MA, USA) and reverse-transcribed to cDNA with a RevertAid First Strand cDNA synthesis kit (K1622, Thermo Fisher Scientific, Waltham, MA, USA). Quantitative real-time PCR was performed on an ABI QuantStudio 1 (A40426, Thermo Fisher Scientific, Waltham, MA, USA) using GoTaq^®^ qPCR Master Mix (A6002, Promega, Madison, WI, USA). The primer sequences are displayed in supplementary Table 3. The results were calculated as 2^−ΔΔct^ values normalized to the expression levels of the housekeeping gene 18 S.

### Statistical analysis

Student’s t test was used for comparisons between two groups, and one-way or two-way ANOVA with Tukey’s multiple comparisons test was used for comparisons among more than two groups. In the t test experiments, an unpaired t test was used when two samples were independent of each other, and a paired t test was used for duplicate or paired samples. One-way ANOVA was used to determine the significance of differences between multiple groups. Two-way ANOVA was performed for pairwise comparisons among multiple groups. *P* < 0.05 was considered to indicate statistical significance. All the statistical analyses were performed with GraphPad Prism 8.0 software (GraphPad Software, Inc., La Jolla, CA, USA). The data are expressed as the means ± standard deviations (SDs).

## Supplementary Information

Below is the link to the electronic supplementary material.

Supplementary Material 1

## Data Availability

The data supporting the results is provided within the manuscript and supplementary information files .

## References

[1] Hak, D. J. et al. Delayed union and nonunions: epidemiology, clinical issues, and financial aspects. *Injury* **45** (Suppl 2), 3–7. 10.1016/j.injury.2014.04.002 (2014). 24857025 10.1016/j.injury.2014.04.002

[2] Wildemann, B. et al. *Non-union Bone Fractures* **7**, 57 (2021). 10.1038/s41572-021-00289-8 34354083

[3] Schmal, H. et al. Nonunion - consensus from the 4th annual meeting of the Danish orthopaedic trauma society. *EFORT Open. Reviews*. **5**, 46–57. 10.1302/2058-5241.5.190037 (2020). 32071773 10.1302/2058-5241.5.190037 PMC7017598

[4] Johnson, L. et al. Physical health and psychological outcomes in adult patients with long-bone fracture non-unions: evidence today. *J. Clin. Med.* **8**10.3390/jcm8111998 (2019). 10.3390/jcm8111998 PMC6912678 31731803

[5] Luby, A. O. et al. Stem cells for bone regeneration: current state and future directions. *J. Craniofac. Surg.* **30**, 730–735. 10.1097/scs.0000000000005250 (2019). 30817525 10.1097/SCS.0000000000005250

[6] Trompet, D., Melis, S., Chagin, A. S. & Maes, C. Skeletal stem and progenitor cells in bone development and repair. *J. Bone Min. Res: Official J. Am. Soc. Bone Min. Res.* **39**, 633–654. 10.1093/jbmr/zjae069 (2024). 10.1093/jbmr/zjae069 38696703

[7] Abdallah, B. M. et al. CD34 defines an osteoprogenitor cell population in mouse bone marrow stromal cells. *Stem Cell. Res.* **15**, 449–458. 10.1016/j.scr.2015.09.005 (2015). 26413784 10.1016/j.scr.2015.09.005

[8] Maacha, S. et al. Paracrine mechanisms of mesenchymal stromal cells in angiogenesis. *Stem Cells Int.* **2020** (4356359). 10.1155/2020/4356359 (2020). 10.1155/2020/4356359 PMC7085399 32215017

[9] Ramasamy, S. K., Kusumbe, A. P., Wang, L. & Adams, R. H. Endothelial Notch activity promotes angiogenesis and osteogenesis in bone. *Nature* **507**, 376–380. 10.1038/nature13146 (2014). 24647000 10.1038/nature13146 PMC4943529

[10] Shen, B. et al. A mechanosensitive peri-arteriolar niche for osteogenesis and lymphopoiesis. *Nature* **591**, 438–444. 10.1038/s41586-021-03298-5 (2021). 33627868 10.1038/s41586-021-03298-5 PMC7979521

[11] Kumar, S., Wan, C., Ramaswamy, G., Clemens, T. L. & Ponnazhagan, S. Mesenchymal stem cells expressing osteogenic and angiogenic factors synergistically enhance bone formation in a mouse model of segmental bone defect. *Mol. Therapy: J. Am. Soc. Gene Therapy*. **18**, 1026–1034. 10.1038/mt.2009.315 (2010). 10.1038/mt.2009.315 PMC2890123 20068549

[12] Li, S. et al. Cell communication and relevant signaling pathways in osteogenesis-angiogenesis coupling. *Bone Res.* **13**10.1038/s41413-025-00417-0 (2025). 10.1038/s41413-025-00417-0 PMC11977258 40195313

[13] Kusumbe, A. P., Ramasamy, S. K. & Adams, R. H. Coupling of angiogenesis and osteogenesis by a specific vessel subtype in bone. *Nature* **507**, 323–328. 10.1038/nature13145 (2014). 24646994 10.1038/nature13145 PMC4943525

[14] Grosso, A. et al. It takes two to tango: coupling of angiogenesis and osteogenesis for bone regeneration. *Front. Bioeng. Biotechnol.* **5**10.3389/fbioe.2017.00068 (2017). 10.3389/fbioe.2017.00068 PMC5675838 29164110

[15] Moya, I. M. & Halder, G. Hippo-YAP/TAZ signalling in organ regeneration and regenerative medicine. *Nat. Rev. Mol. Cell Biol.* **20**, 211–226. 10.1038/s41580-018-0086-y (2019). 30546055 10.1038/s41580-018-0086-y

[16] Wang, X. et al. YAP/TAZ orchestrate VEGF signaling during developmental angiogenesis. *Dev. Cell*. **42**, 462–478e467. 10.1016/j.devcel.2017.08.002 (2017). 28867486 10.1016/j.devcel.2017.08.002

[17] Ong, Y. T. et al. A YAP/TAZ-TEAD signalling module links endothelial nutrient acquisition to angiogenic growth. *Nat. Metabolism*. **4**, 672–682. 10.1038/s42255-022-00584-y (2022). 10.1038/s42255-022-00584-y PMC9236904 35726026

[18] Shen, Y. et al. STAT3-YAP/TAZ signaling in endothelial cells promotes tumor angiogenesis. *Sci. Signal.* **14**, eabj8393. 10.1126/scisignal.abj8393 (2021). 34874746 10.1126/scisignal.abj8393

[19] Sivaraj, K. K. et al. YAP1 and TAZ negatively control bone angiogenesis by limiting hypoxia-inducible factor signaling in endothelial cells. *Elife*. **9**, e50770. https://doi.org/10.7554/eLife.50770 (2020). 10.7554/eLife.50770 PMC6970532 31958058

[20] Ruan, Z. et al. Metformin accelerates bone fracture healing by promoting type H vessel formation through Inhibition of YAP1/TAZ expression. *Bone Res.* **11**10.1038/s41413-023-00279-4 (2023). 10.1038/s41413-023-00279-4 PMC10432554 37587136

[21] Pugh, C. W. & Ratcliffe, P. J. J. N. M. Regulation of angiogenesis by hypoxia: role of the HIF system. *Nat. Med*. **9**, 677–684. https://doi.org/10.1038/nm0603-677 (2003). 10.1038/nm0603-677 12778166

[22] Huang, J. et al. Dietary supplementation of total flavonoids from rhizoma drynariae improves bone health in older caged laying hens. *Poultry Sci*. **99**, 5047–5054. https://doi.org/10.1016/j.psj.2020.06.057 (2020). 10.1016/j.psj.2020.06.057 PMC7598317 32988541

[23] Zhao, Y., Cai, X., Sun, J., Bi, W. & Yu, Y. Active components and mechanisms of total flavonoids from rhizoma drynariae in enhancing cranial bone regeneration: an investigation employing serum pharmacochemistry and network pharmacology approaches. *J. Ethnopharmacol.* **319**, 117253. 10.1016/j.jep.2023.117253 (2024). 37778522 10.1016/j.jep.2023.117253

[24] Li, S. et al. Efficacy of total flavonoids of rhizoma drynariae on the blood vessels and the bone graft in the induced membrane. *Phytomedicine: Int. J. Phytother. Phytopharmacol.* **99**, 153995. 10.1016/j.phymed.2022.153995 (2022). 10.1016/j.phymed.2022.153995 35278899

[25] Li, S. et al. Total flavonoids of rhizoma drynariae promotes differentiation of osteoblasts and growth of bone graft in induced membrane partly by activating Wnt/β-Catenin signaling pathway. *Front. Pharmacol.* **12**, 675470. 10.3389/fphar.2021.675470 (2021). 34122101 10.3389/fphar.2021.675470 PMC8188237

[26] Shen, Z. et al. Total flavonoids of rhizoma drynariae enhances Angiogenic-Osteogenic coupling during distraction osteogenesis by promoting type H vessel formation through PDGF-BB/PDGFR-β instead of HIF-1α/ VEGF axis. *Front. Pharmacol.* **11**, 503524. 10.3389/fphar.2020.503524 (2020). 33328980 10.3389/fphar.2020.503524 PMC7729076

[27] Lin, H. et al. Total flavonoids of rhizoma drynariae promote angiogenesis and osteogenesis in bone defects. *Phytother. Res.* **36**, 3584–3600. 10.1002/ptr.7525 (2022). 35960140 10.1002/ptr.7525

[28] Su, H. et al. Therapeutic potential of total flavonoids of rhizoma drynariae: inhibiting adipogenesis and promoting osteogenesis via MAPK/HIF-1α pathway in primary osteoporosis. *J. Orthop. Surg, Res.* **20**, 260. 10.1186/s13018-025-05665-8 (2025). 40069718 10.1186/s13018-025-05665-8 PMC11895304

[29] Chen, G. Y. et al. Network pharmacology analysis and experimental validation to investigate the mechanism of total flavonoids of rhizoma drynariae in treating rheumatoid arthritis. *Drug. Des. Devel. Ther.* **16**, 1743–1766. 10.2147/dddt.S354946 (2022). 35702063 10.2147/DDDT.S354946 PMC9188779

[30] Long, F. Building strong bones: molecular regulation of the osteoblast lineage. *Nat. Rev. Mol. Cell Biol.* **13**, 27–38. 10.1038/nrm3254 (2012). 10.1038/nrm3254 22189423

[31] Tamma, R., Carbone, C. & Colucci, S. J. I. o. p. j. A. c. r. & perspective, c. Bone matrix proteins and mineralization process. 15–25 (2014).

[32] Zoch, M. L., Clemens, T. L. & Riddle, R. C. New insights into the biology of osteocalcin. *Bone* **82**, 42–49. 10.1016/j.bone.2015.05.046 (2016). 26055108 10.1016/j.bone.2015.05.046 PMC4670816

[33] Xia H. et al. Tissue repair and regeneration with endogenous stem cells. *Nat. Rev. Mater.* **3**,174-193. https://doi.org/10.1038/s41578-018-0027-6 (2018).

[34] Ambrosi, T. H. et al. Aged skeletal stem cells generate an inflammatory degenerative niche. *Nature*. **597**, 256–262. https://doi.org/10.1038/s41586-021-03795-7 (2021). 10.1038/s41586-021-03795-7 PMC8721524 34381212

[35] Duchamp, O. et al. Periosteum contains skeletal stem cells with high bone regenerative potential controlled by Periostin. *Nat. Commun*. **9**, 773. https://doi.org/10.1038/s41467-018-03124-z (2018). 10.1038/s41467-018-03124-z PMC5823889 29472541

[36] Maes, C., Carmeliet, G. & Schipani, E. Hypoxia-driven pathways in bone development, regeneration and disease. *Nat. Rev. Rheumatol.* **8**, 358–366. 10.1038/nrrheum.2012.36 (2012). 22450551 10.1038/nrrheum.2012.36

[37] Shao, J. et al. A dual role of HIF1α in regulating osteogenesis–angiogenesis coupling. *Stem Cell Res Ther*. **13**, 59. https://doi.org/10.1186/s13287-022-02742-1 (2022). 10.1186/s13287-022-02742-1 PMC8818171 35123567

[38] Zhang, Y. et al. Total flavonoids from rhizoma drynariae (Gusuibu) for treating osteoporotic fractures: implication in clinical practice. *Drug. Des. Devel. Ther.* **11**, 1881–1890. 10.2147/dddt.S139804 (2017). 28694688 10.2147/DDDT.S139804 PMC5491704

[39] Wang, L. et al. Prevalence of osteoporosis and fracture in china: the China osteoporosis prevalence study. *JAMA Network Open*. **4**, e2121106. http://doi.org/10.1001/jamanetworkopen.2021.21106 (2021). 10.1001/jamanetworkopen.2021.21106 PMC8369359 34398202

[40] Sun, W. et al. Total flavonoids of rhizoma drynariae ameliorates bone formation and mineralization in BMP-Smad signaling pathway induced large tibial defect rats. *Biomed. pharmacotherapy = Biomedecine Pharmacotherapie*. **138**, 111480. 10.1016/j.biopha.2021.111480 (2021). 10.1016/j.biopha.2021.111480 33774316

[41] Xian, C. J., Zhou, F. H., McCarty, R. C. & Foster, B. K. Intramembranous ossification mechanism for bone bridge formation at the growth plate cartilage injury site. *J. Orthop. Res.: Official Public. Orthop. Res. Soc.* **22**, 417–426. 10.1016/j.orthres.2003.08.003 (2004). 10.1016/j.orthres.2003.08.003 15013105

[42] Liu, C. et al. Mechanical loading promotes the expansion of primitive osteoprogenitors and organizes matrix and vascular morphology in long bone defects. *J. Bone Min. Res.: Official J. Am. Soc. Bone Min. Res.* **34**, 896–910. 10.1002/jbmr.3668 (2019). 10.1002/jbmr.3668 PMC8263903 30645780

[43] Liu, C. et al. Effects of mechanical loading on cortical defect repair using a novel mechanobiological model of bone healing. *Bone*. **108**, 145–155. https://doi.org/10.1016/j.bone.2017.12.027 (2018). 10.1016/j.bone.2017.12.027 PMC8262576 29305998

[44] Wang, Z. et al. Periostin(+) myeloid cells improved long bone regeneration in a mechanosensitive manner. *Bone Res.* **12**, 59. 10.1038/s41413-024-00361-5 (2024). 39406726 10.1038/s41413-024-00361-5 PMC11480347

[45] Mizoguchi, T. & Ono, N. The diverse origin of bone-forming osteoblasts. *J. Bone Min. Res.: Official J. Am. Soc. Bone Min. Res.* **36**, 1432–1447. 10.1002/jbmr.4410 (2021). 10.1002/jbmr.4410 PMC8338797 34213032

[46] Hock, J. M. et al. Osteoblast apoptosis and bone turnover. *J. Bone Min. Res.: Official J. Am. Soc. Bone Min. Res.* **16**, 975–984. 10.1359/jbmr.2001.16.6.975 (2001). 10.1359/jbmr.2001.16.6.975 11393794

[47] Shen, Z. et al. Total flavonoids of rhizoma drynariae enhances CD31hiEmcnhi vessel formation and subsequent bone regeneration in rat models of distraction osteogenesis by activating PDGF-BB/VEGF/RUNX2/OSX signaling axis. *Int. J. Mol. Med*. **50**, 112. https://doi.org/10.3892/ijmm.2022.5167 (2022). 10.3892/ijmm.2022.5167 PMC9330352 35795995

[48] Filipowska, J., Tomaszewski, K. A., Niedźwiedzki, Ł., Walocha, J. A. & Niedźwiedzki, T. The role of vasculature in bone development, regeneration and proper systemic functioning. *Angiogenesis* **20**, 291–302. 10.1007/s10456-017-9541-1 (2017). 28194536 10.1007/s10456-017-9541-1 PMC5511612

[49] Chen, J. et al. Gli1(+) cells couple with type H vessels and are required for type H vessel formation. *Stem Cell. Rep.* **15**, 110–124. 10.1016/j.stemcr.2020.06.007 (2020). 10.1016/j.stemcr.2020.06.007 PMC7363988 32668219

[50] Fang, T. D. et al. Angiogenesis is required for successful bone induction during distraction osteogenesis. *J. Bone Min. Res.: Official J. Am. Soc. Bone Min. Res.* **20**, 1114–1124. 10.1359/jbmr.050301 (2005). 10.1359/JBMR.050301 15940364

[51] Kim, J. et al. YAP/TAZ regulates sprouting angiogenesis and vascular barrier maturation. *J. Clin. Invest*. **127**, 3441–3461. http://doi.org/10.1172/JCI93825 (2017). 10.1172/JCI93825 PMC5669570 28805663

[52] Yasuda, D. et al. Lysophosphatidic acid-induced YAP/TAZ activation promotes developmental angiogenesis by repressing Notch ligand Dll4. *J. Clin. Investig.* **129**, 4332–4349. 10.1172/jci121955 (2019). 31335323 10.1172/JCI121955 PMC6763231

[53] Astone, M. et al. Zebrafish mutants and TEAD reporters reveal essential functions for Yap and Taz in posterior Cardinal vein development. *Sci. Rep.* **8**, 10189. 10.1038/s41598-018-27657-x (2018). 29976931 10.1038/s41598-018-27657-x PMC6033906

[54] Sivaraj, K. K. et al. YAP1 and TAZ negatively control bone angiogenesis by limiting hypoxia-inducible factor signaling in endothelial cells. *eLife* **9**10.7554/eLife.50770 (2020). 10.7554/eLife.50770 PMC6970532 31958058

[55] Ester, L. et al. Transcriptional regulators YAP and TAZ have distinct abilities to compensate for one another in podocytes. *J. Am. Soc. Nephrol.: JASN*. **36**, 1520–1534. 10.1681/asn.0000000689 (2025). 40137583 10.1681/ASN.0000000689 PMC12352792

[56] Manshori, M. et al. Greater angiogenic and immunoregulatory potency of bFGF and 5-aza-2ʹ-deoxycytidine pre-treated menstrual blood stem cells in compare to bone marrow stem cells in rat model of myocardial infarction. *BMC Cardiovasc. Disord*. **22**, 578. https://doi.org/10.1186/s12872-022-03032-7 (2022). 10.1186/s12872-022-03032-7 PMC9805241 36587199

[57] Han, L., Wang, C., Wang, T., Hu, Y. & Wang, H. J. E. T. Total flavonoids of rhizoma drynariae improves tendon-bone healing for anterior cruciate ligament reconstruction in mice and promotes the osteogenic differentiation of bone mesenchymal stem cells by the ERR1/2‐Gga1‐TGF‐β/MAPK pathway. *Environ. Toxicol*. **39**, 106–119. https://doi.org/10.1002/tox.23955 (2024). 10.1002/tox.23955 37665165

[58] Yao, W. et al. Effect of total flavonoids of rhizoma drynariae on tibial dyschondroplasia by regulating BMP-2 and Runx2 expression in chickens. *Front. Pharmacol*. **9**, 1251. https://doi.org/10.3389/fphar.2018.01251 (2018). 10.3389/fphar.2018.01251 PMC6224448 30450047

[59] Soleimani, M. & Nadri, S. A protocol for isolation and culture of mesenchymal stem cells from mouse bone marrow. *Nat. Protoc.* **4**, 102–106. 10.1038/nprot.2008.221 (2009). 19131962 10.1038/nprot.2008.221

