# Supplementary material for: Total flavonoids of rhizoma drynariae improved the osteogenic and pro-angiogenic capacities of BMSCs to accelerate long bone regeneration
Source: Sci Rep. 2025 Oct 10;15:35547. doi: 10.1038/s41598-025-19511-8 (PMC12514199; doi:10.1038/s41598-025-19511-8)
Supplement: Supplementary file 1 — Supplementary Material 1 [file 41598_2025_19511_MOESM1_ESM.docx]

**Supplementary Information for**

**Total Flavonoids of Rhizoma Drynariae Improved the Osteogenic and Pro-angiogenic Capacities of BMSCs to** **Accelerate Long bone regeneration**

**Hengjun Huang ^1,2^, Silu Li ^1,2^, Huang Zhan ^1,2^, Fenfang Gong ^1,2^, Jian Liu ^1,2^, Zhenya Liu ^1,2^, Hui Li ^1,2,3 *^ and Chengyu Yang ^1,2,*^**

^1^ Jiangxi Province Key Laboratory of Traditional Chinese Medicine Pharmacology, Institute of Traditional Chinese Medicine Health Industry, China Academy of Chinese Medical Sciences, Nanchang 330115, China;

^2^ Jiangxi Health Industry Institute of Traditional Chinese Medicine, Nanchang 330115, China;

^3^ Institute of Chinese Materia Medica, China Academy of Chinese Medical Sciences, Beijing 100700, China;

* Correspondence: 11930896@mail.sustech.edu.cn (C.Y.); lihuiyiren@ 163.com (H.L.)

# These authors contributed equally to this work.

**This file includes:**

Supplementary Fig. 1. UPLC chromatograms of TFRD in (A) negative-ion mode and (B) positive-ion mode

Supplementary Table 1. Characterization of TFRD by UPLC-MS

Supplementary Fig. 2. Secondary mass spectrum and structural formula of major components in TFRD

Supplementary Fig. 3. Flow cytometry analysis for the identification of isolated cells

Supplementary Fig. 4. QPCR analysis of the inhibitory efﬁciency of siRNAs targeting YAP1 or TAZ

Supplementary Table 2. Target sequences of siRNAs

Supplementary Table 3. The primer sequences for q-PCR

Supplementary Fig.5. Representative imaging of the negative control for immunofluorescence staining


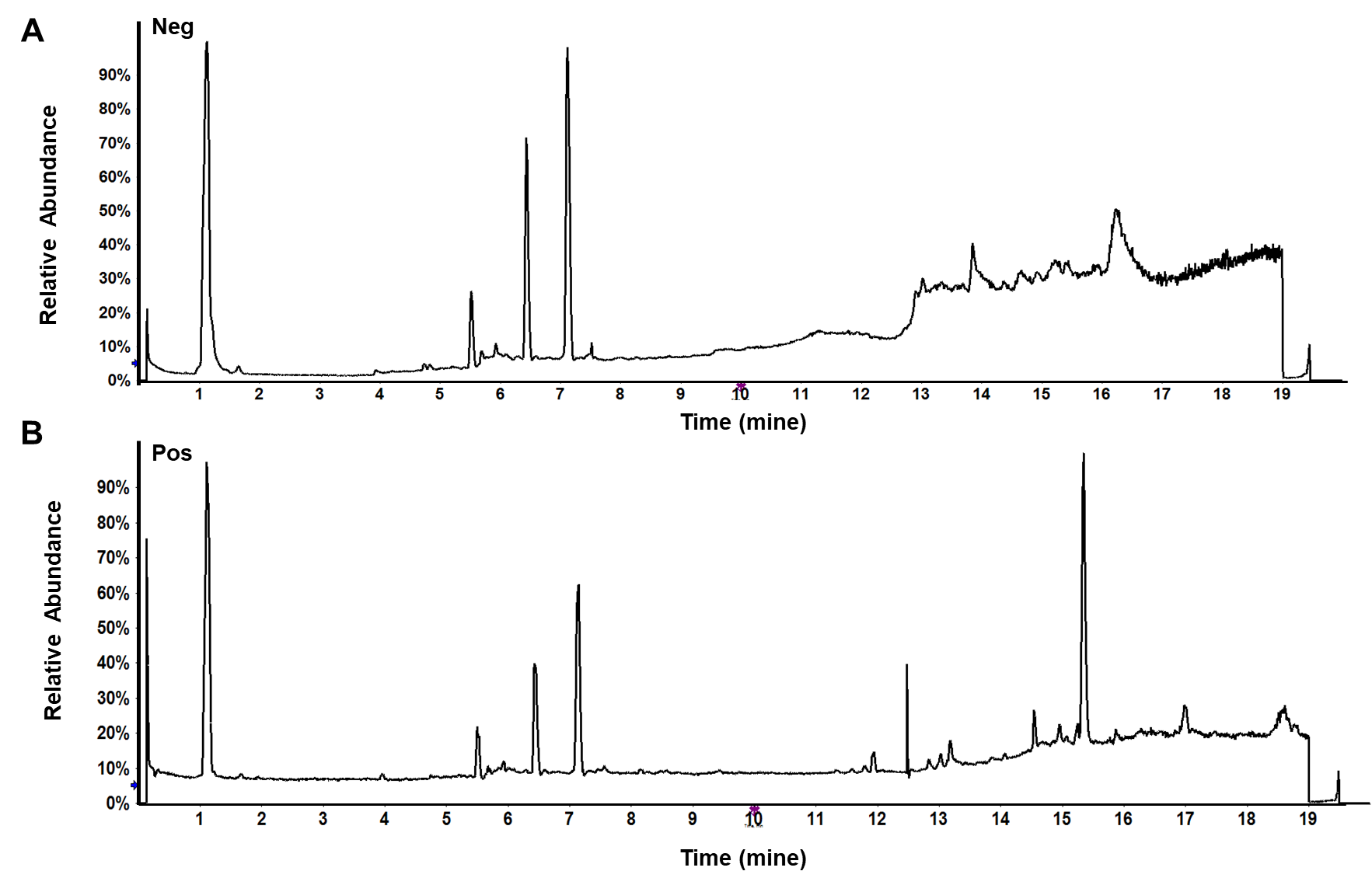


**Supplementary Fig. 1**. **UPLC-MS chromatograms of TFRD in (A) negative-ion mode and (B) positive-ion mode**

**Supplementary Table 1.** **Characterization of TFRD by UPLC-MS**

| No | Compound | Formula | Retention time (min) | Ion | Error(ppm) | MS/MS |
| --- | --- | --- | --- | --- | --- | --- |
| 1 | Davallioside A_qt | C_25_H_29_NO_12_ | 5.67 | M-H | -1.2 | 503.1062,  415.0357 |
| 2 | Kaempferol | C_15_H_10_O_6_ | 5.99 | M-H | -0.2 | 285.0383  185.0651 |
| 3 | Naringenin-7-O-glucopyranoside | C_21_H_22_O_10_ | 6.25 | M-H | 0.8 | 271.0596,  151.0030 |
| 4 | (+)-Catechin | C_15_H_14_O_6_ | 6.44 | M+H | -2.6 | 289.0695, 109.0287 |
| 5 | Neoeriocitrin | C_27_H_32_O_15_ | 6.44 | M-H | -1.2 | 271.0579, 151.0014 |
| 6 | Xanthogalenol | C_21_H_22_O_5_ | 6.78 | M-H | -9.4 | 355.0551, 179.0872 |
| 7 | Eriodyctiol | C_15_H_12_O_6_ | 6.59 | M-H | -1 | 151.0024, 135.0433 |
| 8 | Naringin | C_27_H_32_O_14_ | 7.12 | M-H | -1.2 | 271.0579, 151.0014 |
| 10 | Naringenin | C_15_H_12_O_5_ | 7.44 | M-H | -0.7 | 271.0579, 151.0014 |
| 11 | Baicalein | C_15_H_10_O_5_ | 7.44 | M+H | 2.1 | 233.0476, 171.0667 |
| 12 | Afzelin | C_21_H_20_O_10_ | 8.06 | M-H | -0.9 | 285.0375, 255.0723 |
| 13 | Neohesperidin | C_28_H_34_O_15_ | 12.47 | M+H | -1.1 | 611.1608, 303.0827 |

**
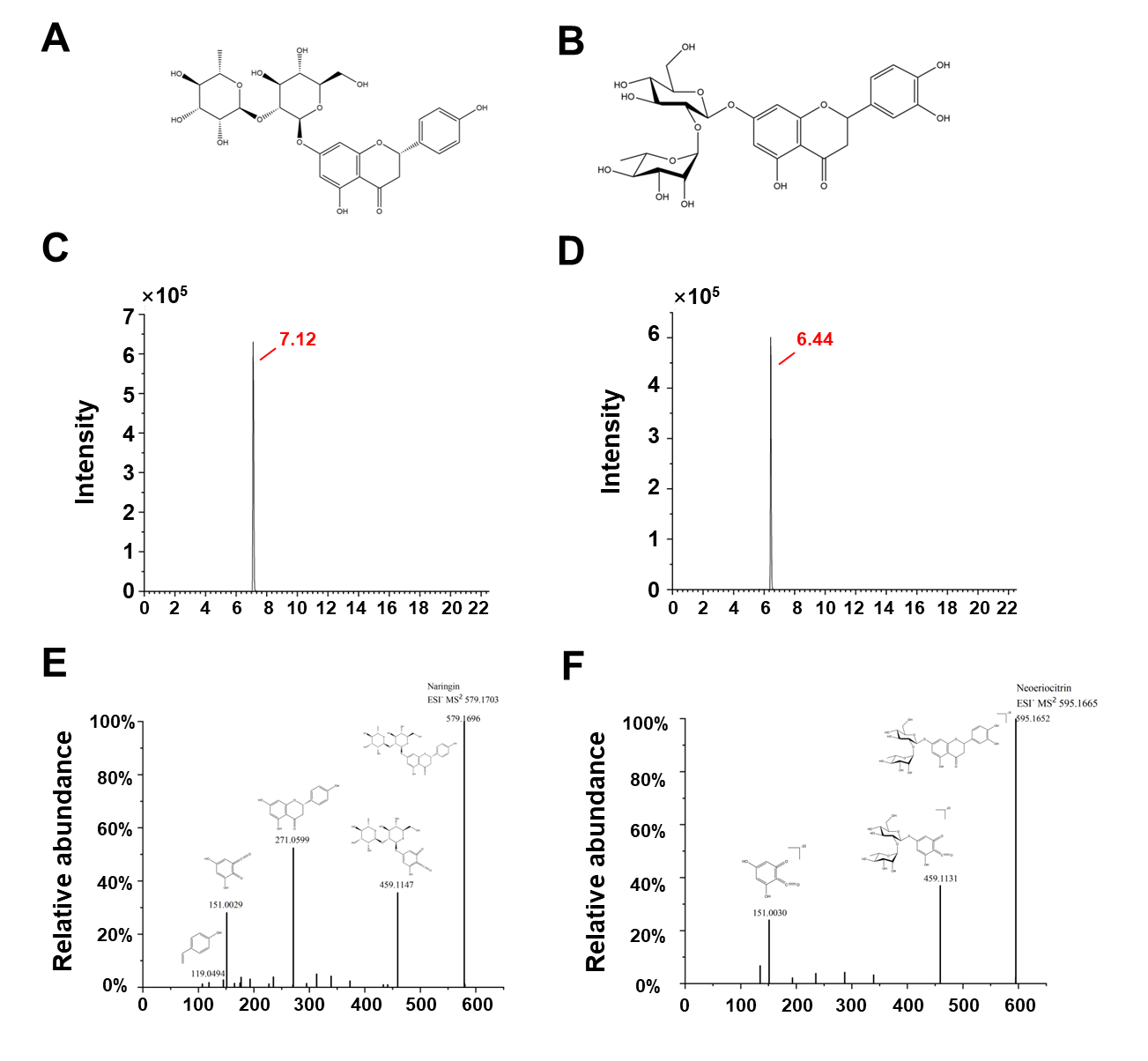
**

**Supplementary Fig. 2.** The structural formula of **(A)** naringin **(B)** and neoeriocitrin. The primary mass spectrum of **(C)** naringin and **(D)** neoeriocitrin in TFRD**.** The secondary mass spectrum of **(E)** naringin and **(F)** neoeriocitrin in TFRD.


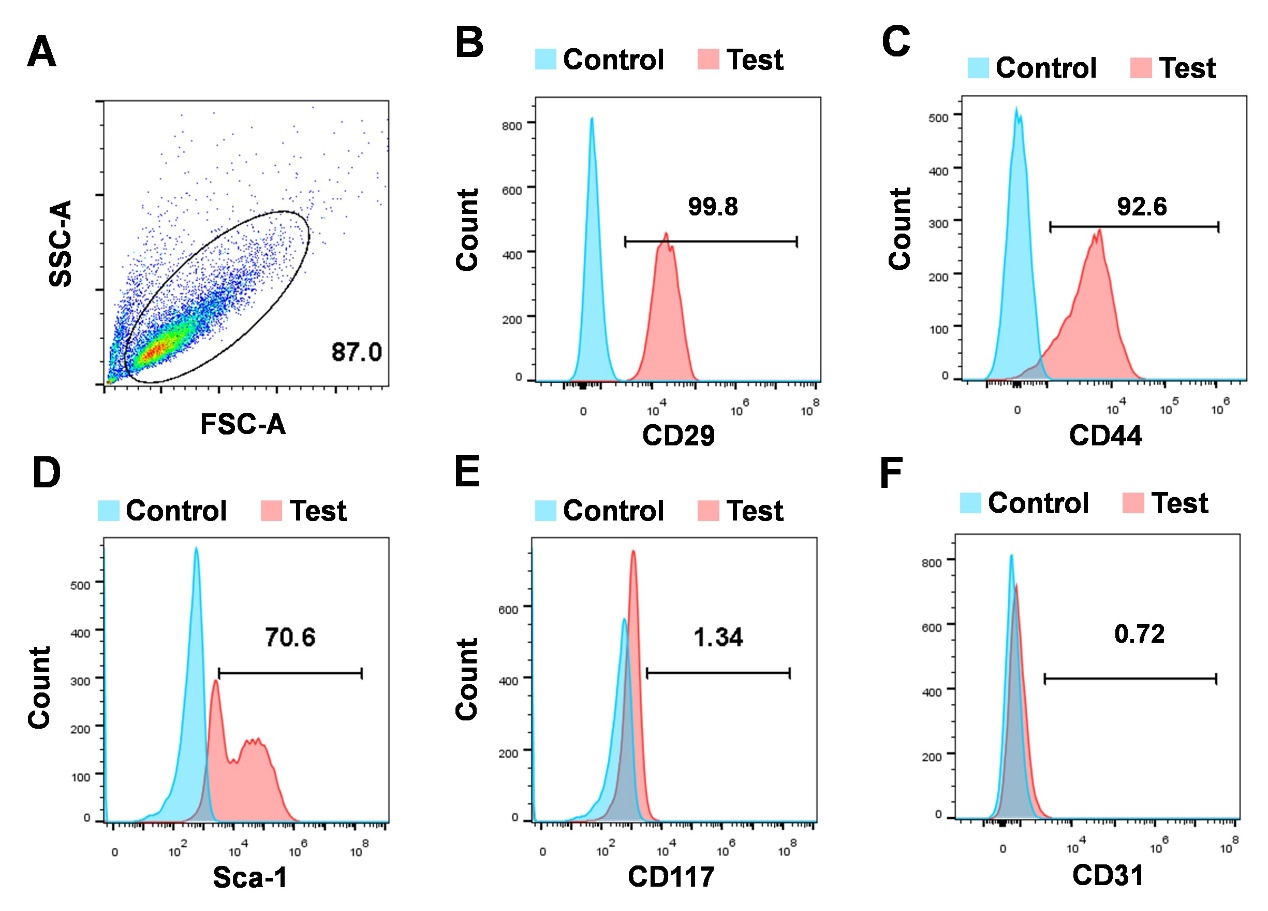


**Supplementary Fig. 3. Flow cytometry analysis for the identification of isolated cells. (A)** Gating strategy and representing of **(B)** CD29, **(C)** CD44, **(D)** Sca-1, **(E)** CD117 and (F) CD31 in isolated cells by utilizing flow cytometry. Control: isolated cells without antibody labeling; Test: isolated cells with antibody labeling


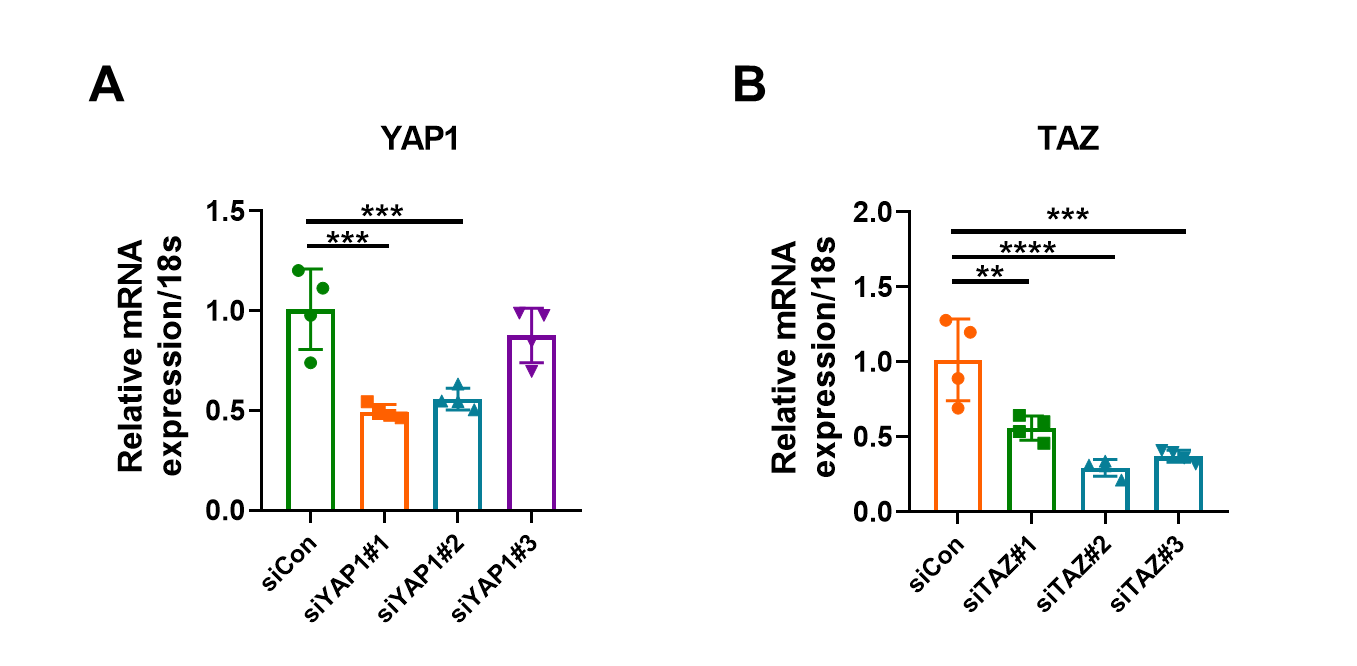


**Supplementary Fig. 4. QPCR analysis of the inhibitory efﬁciency of siRNAs targeting YAP1 or TAZ.** The relative expression level of **(A)** YAP1 and **(B)** TAZ in HUVECs after 48h of siRNA treatment (n=4). *: P<0.05; **:P<0.01; ***: P<0.001; ****: P<0.0001.

**Supplementary Table 2. Target sequences of siRNAs**

| siRNA | Target sequences (5'-3') |
| --- | --- |
| si-YAP1#1 | GAGATGGAATGAACATAGA |
| Si-YAP1#2 | CCACCAAGCTAGATAAAGA |
| Si-YAP1#3 | GTAGCCAGTTACCAACACT |
| Si-TAZ #1 | CGATGAATCAGCCTCTGAA |
| Si-TAZ #2 | AGAGTCTGCTCTGAACAAA |
| Si-TAZ #3 | GGACAAACACCCATGAACA |

**Supplementary Table 3. The primer sequences for q-PCR**

| Primer name | Sequences (5'-3') |
| --- | --- |
| ALP F | GGACAGGACACACACACACA |
| ALP R | CAAACAGGAGAGCCACTTCA |
| RUNX2 F | TCCCTGAACTCTGCACCAAG |
| RUNX2 R | ATCTGGCTCAGGTAGGAGGG |
| OCN F | CTGACAAAGCCTTCATGTCCAA |
| OCN R | GCGCCGGAGTCTGTTCACTA |
| Col1 F | AGAGCGGTGAGTCTAAGGAGT |
| Col1 R | TGCCCTTTCCGTTGTTGTCC |
| VEGFA-F | GCACCCATGGCAGAAGGAGG |
| VEGFA-R | CCTTGGTGAGGTTTGATCCGCATA |
| Hif-1α-F | TCAAGTCAGCAACGTGGAAG |
| Hif-1α-R | TATCGAGGCTGTGTCGACTG |
| VEGFR2-F | AGCTGAATCACCCAGAGTCC |
| VEGFR2-R | TGCAATCAATAGAAGGAACACG |
| bFGF-F | GGAGAAGAGCGACCCTCACATCAAG |
| bFGF-R | CCAGTTCGTTTCAGTGCCACATACCAA |
| YAP1-F | GAACTGCTTCGGCAGGTGAG |
| YAP1-R | GCAGGGCTAACTCCTGACATT |
| TAZ-F | TCACATCCTGGCGACTCTCA |
| TAZ-R | GAGGCCGGATTCATCTTCTGG |
| CTGF-F | GAAGCTGACCTGGAGGAAAA |
| CTGF-R | ACTGGCAGAGTGGTGGTTCT |
| CYR61-F | GCTCTAGAATGAGCTCCCGCATCGCCA |
| CYR61-R | GGAATTCTTAGTCCCTAAATTTGTGA |
| 18S_1 F | GAGAAACGGCTACCACATCC |
| 18S_1 R | CCTCCAATGGATCCTCGTTA |

**
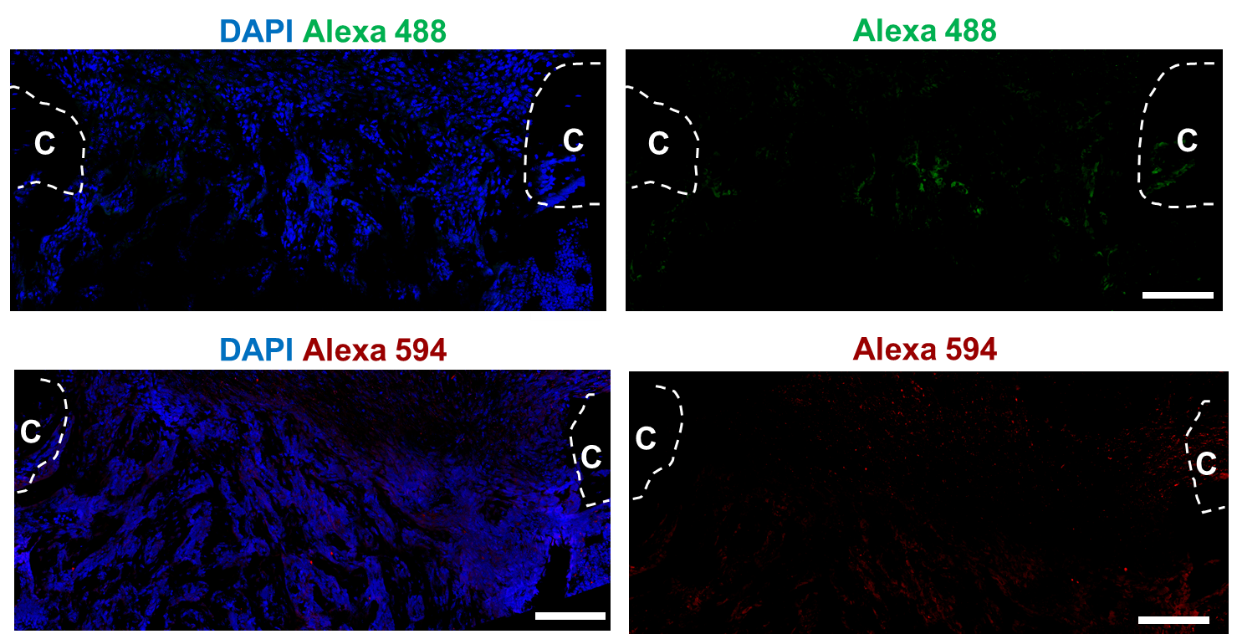
**

**Supplementary Fig. 5. Representative imaging of the negative control for immunofluorescence staining.** Negative control: Primary antibody omission (secondary antibody only)
